# Essential Oils as Antiviral Agents, Potential of Essential Oils to Treat SARS-CoV-2 Infection: An In-Silico Investigation

**DOI:** 10.3390/ijms21103426

**Published:** 2020-05-12

**Authors:** Joyce Kelly R. da Silva, Pablo Luis Baia Figueiredo, Kendall G. Byler, William N. Setzer

**Affiliations:** 1 Laboratório de Biotecnologia de Enzimas e Biotransformações, Universidade Federal do Pará, Belém PA 66075-900, Brazil; joycekellys@ufpa.br; 2 Departamento de Ciências Naturais, Centro de Ciências Sociais e Educação, Universidade do Estado do Pará, Belém PA 66050-540, Brazil; pablo.figueiredo@uepa.br; 3 Department of Biological Sciences, University of Alabama in Huntsville, Huntsville, AL 35899, USA; kgb0011@uah.edu; 4 Department of Chemistry, University of Alabama in Huntsville, Huntsville, AL 35899, USA; 5 Aromatic Plant Research Center, 230 N 1200 E, Suite 100, Lehi, UT 84043, USA

**Keywords:** COVID-19, corona virus, molecular docking, antiviral, essential oils

## Abstract

Essential oils have shown promise as antiviral agents against several pathogenic viruses. In this work we hypothesized that essential oil components may interact with key protein targets of the 2019 severe acute respiratory syndrome coronavirus 2 (SARS-CoV-2). A molecular docking analysis was carried out using 171 essential oil components with SARS-CoV-2 main protease (SARS-CoV-2 M^pro^), SARS-CoV-2 endoribonucleoase (SARS-CoV-2 Nsp15/NendoU), SARS-CoV-2 ADP-ribose-1″-phosphatase (SARS-CoV-2 ADRP), SARS-CoV-2 RNA-dependent RNA polymerase (SARS-CoV-2 RdRp), the binding domain of the SARS-CoV-2 spike protein (SARS-CoV-2 rS), and human angiotensin−converting enzyme (hACE2). The compound with the best normalized docking score to SARS-CoV-2 M^pro^ was the sesquiterpene hydrocarbon (*E*)-β-farnesene. The best docking ligands for SARS−CoV Nsp15/NendoU were (*E*,*E*)-α-farnesene, (*E*)-β-farnesene, and (*E*,*E*)−farnesol. (*E*,*E*)−Farnesol showed the most exothermic docking to SARS-CoV-2 ADRP. Unfortunately, the docking energies of (*E*,*E*)−α-farnesene, (*E*)-β-farnesene, and (*E*,*E*)−farnesol with SARS-CoV-2 targets were relatively weak compared to docking energies with other proteins and are, therefore, unlikely to interact with the virus targets. However, essential oil components may act synergistically, essential oils may potentiate other antiviral agents, or they may provide some relief of COVID-19 symptoms.

## 1. Introduction

The 2019 severe acute respiratory syndrome coronavirus 2 (SARS-CoV-2) is a newly emerging respiratory illness. The epidemic started in December 2019 in Wuhan, China, and has rapidly spread throughout China and the world and is now a global pandemic. SARS-CoV-2 can be efficiently transmitted among humans and has shown a high degree of morbidity and mortality [1,2]. As of April 20, 2020, the worldwide number of infected individuals was 2,544,792, with as many as 175,694 deaths [3]. There are currently no approved vaccines available for the prevention of SARS-CoV-2 infection and only just recently, remdesivir has received “emergency use authorization” for treatment of COVID-19 in the United States; therefore, there is an urgent demand for potential chemotherapeutic agents to treat this disease.

Essential oils have been screened against several pathogenic viruses (Table 1), including influenza and other respiratory viral infections. Influenza is an infectious respiratory disease caused by one of three types of influenza viruses, type A, type B, or type C [4]. The most significant in terms of human morbidity and mortality is influenza virus type A, which is found in several bird and mammal species [5]. Several different serotypes of influenza type A have caused global flu pandemics [6]: H1N1, which caused the Spanish flu in 1918 (40–50 million deaths worldwide) [7] and the swine flu in 2009 [8]; the Asian flu of 1957–1958 (ca. 1.5 million deaths worldwide) was caused by influenza A H2N2 [8]; serotype H3N2 caused the Hong Kong flu in 1968 [9]; and H5N1, which caused the bird flu in 2004 [10]. Influenza virus type B, however, is largely confined to human hosts [11].

One study evaluated the in vitro antiviral effect against influenza type A (H1N1) of commercial essential oils that included cinnamon (*Cinnamomum zeylanicum*), bergamot (*Citrus bergamia*), lemongrass (*Cymbopogon flexuosus*), thyme (*Thymus vulgaris*), and lavender (*Lavandula angustifolia*). The oils were tested in the liquid phase at a concentration of 0.3% and in the vapor phase. The oils of cinnamon, bergamot, thyme, and lemongrass displayed 100% inhibition of H1N1 in the liquid phase, while the inhibition for lavender essential oil was 85%. However, in the vapor phase, 100% inhibition was observed only for cinnamon leaf essential oil after 30 min of exposure. The bergamot, lemongrass, thyme, and lavender essential oils displayed inhibition rates of 95%, 90%, 70%, and 80%, respectively [12].

*Cinnamomum zeylanicum* leaf oil is characterized by eugenol (75–85%), followed by smaller amounts of linalool (1.6–8.5%), and benzyl benzoate (0.1–8.3%) [13,14,15]. Bergamot oil is rich in limonene (23–55%), linalool (2–37%), and linalyl acetate (12–41%), with lesser quantities of β-pinene (up to 10%) and γ-terpinene (up to 10%) [16,17,18,19,20]. Geranial (48–54%) and neral (29–33%) have been reported as the major components of *C. flexuosus,* but many chemotypes, cultivars, and variants have been reported for *C. flexuosus* [21,22].

In the literature, there have been at least 20 different chemotypes identified for thyme essential oil. The “typical” thyme essential oil presents a thymol content of 45% (range 31–50%), with significant concentrations of *p*-cymene (0.1–26.6%, average = 15.6%) and γ-terpinene (up to 22.8%, average = 9.3%). In addition, there are several other chemotypes of *T. vulgaris* rich in thymol and/or carvacrol [23]. Thymol has been identified as an anti-influenza agent against influenza type A and parainfluenza type 3 virus [24,25]. *Lavandula angustifolia* essential oil is rich in linalyl acetate (37.0–43.6%), linalool (19.7–39.1%), geraniol (up to 9.3%), β-caryophyllene (up to 5.1%), terpinen-4-ol (up to 14.9%), lavandulyl acetate (up to 5.5%), and borneol (up to 6.4%) [26,27,28,29].

Another essential oil with notable anti-influenza effects is tea tree, which is extracted from the leaves of *Melaleuca alternifolia* (Myrtaceae). Commercial tea tree oil is composed of terpinen-4-ol (30–48%), γ-terpinene (10–28%), α-terpinene (5–13%), 1,8-cineole (up to 15%), terpinolene (1.5–5%), *p*-cymene (0.5–12%), α-pinene (1–6%), and α-terpineol (1.5–8%) [30]. Tea tree oil showed 100% inhibition of influenza type A (H1N1) virus at 0.01% concentration and a median inhibitory concentration (IC_50_) of 6 μg/mL [31,32]. In addition, 30 min exposure of type A (H11N9) virus to tea tree oil vapor caused 100% inhibition [33]. The tea tree oil components, terpinen-4-ol, terpinolene, and α-terpineol, have shown anti-influenza virus activity against type A (H1N1), with IC_50_ values of 25, 12, and 250 μg/mL, respectively. α-Terpinene, γ-terpinene, and *p*-cymene were inactive, however [31].

Avian influenza viruses (H5N1) exhibit both high and low virulence in numerous mammalian species, highlighting the connection between the route of inoculation and virus pathogenicity [34]. Since 2003, there have been over 600 documented cases of human infection with H5N1 viruses, with most cases among young, previously healthy individuals [35]. The essential oils extracted from *Citrus reshni* leaves and peel (unripe and ripe fruits) were tested against H5N1 virus by plaque reduction assay. The oils showed moderate inhibition of the H5N1 virus at a concentration of 2.5 μL/mL. Sabinene (40.5%), linalool (23.3%), and terpinen-4-ol (8.3%) were the main constituents in the leaf oil while limonene (82.4%, 91.6%) was the main compound in the fruit peel essential oils (unripe and ripe, respectively) [36].

The essential oil of leaves of *Fortunella margarita* is rich in the sesquiterpenoids β-eudesmol (28.3%), α-muurolene (10.3%), β-gurjunene (10.0%), γ-eudesmol (8.4%), and γ-muurolene (6.6%) while the essential oil extracted from the fruits showed monterpenoids as the main components, α-terpineol (55.5%), carvone (5.7%), and carveol (5.5%). Both samples were tested for antiviral activity against avian influenza (H5N1) virus, and the obtained results revealed that the fruit essential oil was more effective (80% virus inhibition by the MTT (3-(4,5-dimethylthiazol-2-yl)-2,5-diphenyltetrazolium bromide) assay using Madin−Darby canine kidney (MDCK) cells for virus propagation). The IC_50_ values obtained for the leaf and fruit essential oils were 38.89 and 6.77 μg/mL, respectively [53].

Dengue fever, a mosquito−borne disease, is caused by dengue virus (DENV) which includes four major serotypes (DENV-1, -2, -3, and -4). Some serotypes cause more severe diseases than others; severe dengue is associated with secondary infections by a different serotype. *Dengue* disease is a major public health problem in developing tropical countries and has being continuously spreading to new geographical areas [92]. The essential oils of two species of *Lippia* were assayed against four dengue serotypes (DENV-1, DENV-2, DENV-3, DENV-4) [61]. The IC_50_ values for *Lippia alba* oil, rich in carvone (39.7%), limonene (30.6%), and bicyclosesquiphellandrene (8.9%), were between 0.4 and 32.6 μg/mL. However, the *Lippia citrodora* essential oil, composed of geranial (18.9%), neral (15.6%), limonene (10.7%), and 1,8-cineole (5.0%), showed the best activity, with IC_50_ values varying from 1.9 to 33.7 μg/mL. No viral inhibitory effect was observed by addition of the essential oil after virus adsorption; the inhibitory effect of the essential oil seemed to cause direct virus inactivation before adsorption on the host cell.

The essential oils of seven aromatic plants from Córdoba, San Luis, and San Juan provinces (Argentina) were screened for cytotoxicity and in vitro inhibitory activity against dengue virus type 2 (DENV−2) [38]. The oils of *Jungia polita* and *Buddleja cordobensis* were composed of caryophyllene oxide (9.18%, 32.1%) and β-caryophyllene (8.13%, 16.5%) as the major compounds. However, these oils displayed different IC_50_ values (86.4 and 39.8 μg/mL, respectively). The other samples were composed mostly of monoterpenes and displayed lower activity, except *Pectis odorata* oil, which presented limonene (50.2%), neral (27.2%), and geranial (23.6%) as the major compounds and an IC_50_ value of 39.6 μg/mL. In addition, the essential oils of *Artemisia mendozana*, rich in camphor (22.4%), artemisole (11.7%), and artemisia alcohol (10.8%); *Gailardia megapotamica* composed of β-pinene (35.5%), spathulenol (10.7%), and germacrene D (6.8%); and *Heterothalamus alienus* characterized by β-pinene (35.5%), spathulenol (10.7%), and germacrene D (6.8%), showed an average IC_50_ value of 130.63 μg/mL.

Yellow fever (YF), caused by yellow fever virus (YFV), has historically been considered one of the most dangerous infectious diseases. YFV is transmitted to humans via mosquitoes of the *Haemogogus*, *Sabethes*, and *Aedes* genera. Annually, there are approximately 80,000–200,000 YFV cases worldwide, with a case fatality rate (CFR) ranging from 20–60% [93,94]. Essential oils of *Lippia* species and their main compounds have been tested against yellow fever virus (YFV) in Vero cells. The oil of *Lippia origanoides* showed carvacrol (44.0%), thymol (15.0%), and γ-terpinene (10.0%) as the main compounds and displayed 100% inhibition at a concentration of 11.1 μg/mL [43]. However, in the same study, the oil of *L. alba* displayed 100% inhibition at a concentration of 100.0 μg/mL. The major compounds were carvone (51.0%), limonene (33.0%), and bicyclosesquiphellandrene (7.0%). The essential oil of *L. alba* with a similar chemical composition, carvone (39.7%), limonene (30.6%), and bicyclosesquiphellandrene (8.9%), displayed an IC_50_ value of 4.3 μg/mL against YFV when tested in Vero cells using the MTT assay [62]. The essential oil of *L. citriodora*, dominated by geranial (18.9%), neral (15.6%), and limonene (10.7%), did not display a statistical difference in comparison to citral, with IC_50_ values of 19.4 and 17.6 μg/mL, respectively [62].

In addition to essential oils, several individual essential oil components have been screened for antiviral activity (Table 2).

Because of the activities of several essential oils and essential oil components against human pathogenic viruses, we hypothesized that essential oil components may be potentially useful as antiviral agents against SARS-CoV-2. In this work, we carried out a molecular docking analysis of the major components of essential oils that exhibit antiviral activity (Table 1 and Table 2) with known SARS-CoV-2 protein targets.

## 2. Results and Discussion

Several proteins have been identified for severe acute respiratory syndrome coronavirus 2 (SARS-CoV-2), which may serve as potential targets for chemotherapeutic intervention in coronavirus disease 2019 (COVID-19). These protein targets include SARS-CoV-2 main protease (SARS-CoV-2 M^pro^), SARS-CoV-2 endoribonucleoase (SARS-CoV-2 Nsp15/NendoU), SARS-CoV-2 ADP−ribose−1″−phosphatase (SARS-CoV-2 ADRP), SARS-CoV-2 RNA-dependent RNA polymerase (SARS-CoV-2 RdRp), the binding domain of the SARS-CoV-2 spike protein (SARS-CoV-2 rS), and human angiotensin−converting enzyme (hACE2). There have already been several molecular docking studies on these macromolecular targets. Several groups have carried out molecular docking of natural product libraries with SARS-CoV-2 M^pro^ [102,103,104,105]. Additionally, commercially available drugs have also been examined using in silico methods [106,107].

A molecular docking study was carried out with 171 essential oil components with SARS-CoV-2 M^pro^ (PDB: 5R7Z, 5R80, 5R81, 5R82, 5R83, 5R84, 6LU7, 6M03, and 6Y84), SARS-CoV-2 Nsp15/NendoU (PDB: 6VWW, 6W01, and 6W02), SARS-CoV-2 rS (PDB: 6M0J, 6M17, 6VX1, and 6VW1), and SARS-CoV-2 RdRp (PDB: 6M71). The best docking scores are summarized in Table 3.

**Figure 1:**
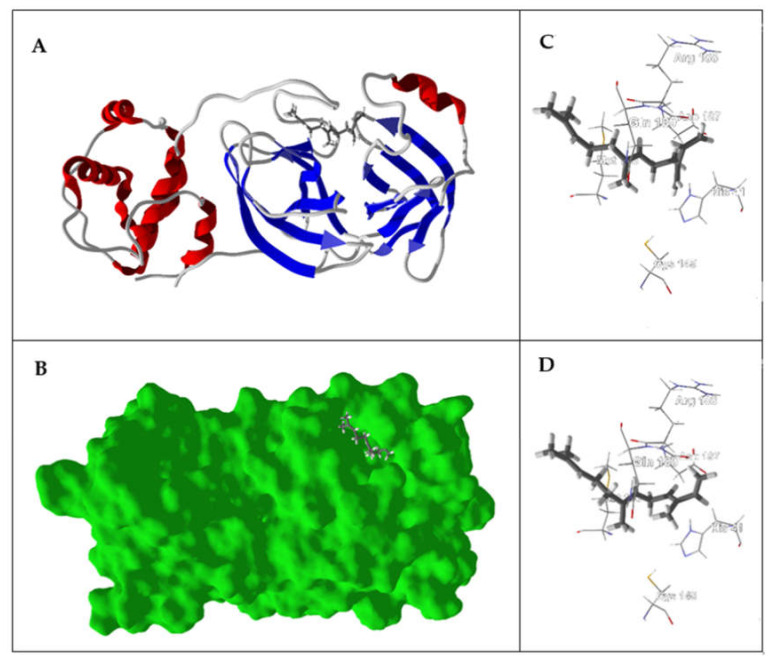
Lowest-energy docked poses of (*E*)-β-farnesene and (*E*,*E*)-α-farnesene with the SARS-CoV-2 main protease (PDB: 6LU7). (**A**) Ribbon structure of the enzyme and (*E*)-β-farnesene. (**B**) Solid structure of the enzyme showing (*E*)-β-farnesene in the binding cleft. (**C**) Amino acid residues in proximity to the docked (*E*)-β-farnesene. (**D**) Lowest-energy docked pose of (*E*,*E*)-α-farnesene in the enzyme binding site.

**Figure 2:**
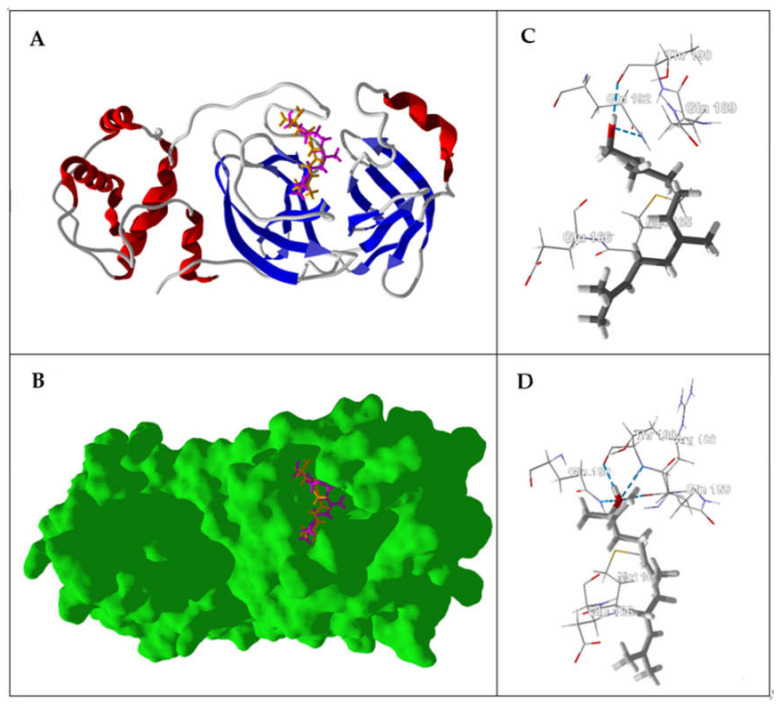
Lowest-energy docked poses of (*E*,*E*)-farnesol and (*E*)-nerolidol with the SARS-CoV-2 main protease (PDB: 6Y84). (**A**) Ribbon structure of the enzyme and (*E*,*E*)-farnesol (magenta) and (*E*)-nerolidol (orange). (**B**) Solid structure of the enzyme showing (*E*,*E*)-farnesol (magenta) and (*E*)-nerolidol (orange) in the binding cleft. (**C**) Important interactions of amino acid residues with (*E*,*E*)-farnesol. (**D**) Important interactions of amino acid residues with (*E*)-nerolidol. Hydrogen bonds are indicated with blue dashed lines.

The main protease, SARS-CoV-2 M^pro^, is a cysteine protease that is essential for processing the polyproteins that are translated from the coronavirus RNA [108]. The substrate binding site of the enzyme is a cleft flanked by Gln189, Met49, Pro168, Glu166 and His41; the active site is Cys145 and His41. The compound with the best normalized docking score to SARS-CoV-2 M^pro^ was the sesquiterpene hydrocarbon (*E*)-β-farnesene (DS_norm_ = −115.4 kJ/mol). Other essential oil components showing good docking scores with SARS-CoV-2 M^pro^ were (*E*,*E*)-α-farnesene (DS_norm_ = −115.0 kJ/mol), (*E*,*E*)-farnesol (DS_norm_ = −112.4 kJ/mol), and (*E*)-nerolidol (DS_norm_ = −110.7 kJ/mol). The sesquiterpene hydrocarbons (*E*,*E*)-α-farnesene and (*E*)-β-farnesene occupy the substrate binding site, flanked by Gln189, Arg188, Met165, His41, and Asp 187 (Figure 1). The lowest-energy docked poses of both (*E*,*E*)-farnesol and (*E*)-nerolidol showed hydrogen bonding of the alcohol moiety to Gln192 and Thr190 and, in the case of (*E*)-nerolidol, also with GLN189 and ARG188 (Figure 2).

**Figure 3:**
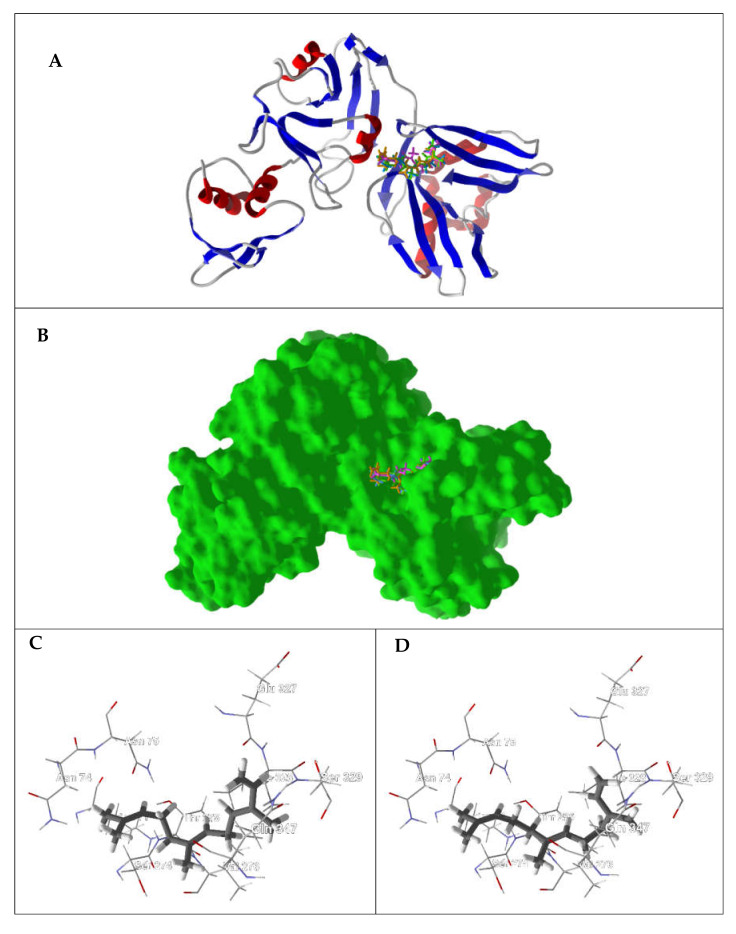

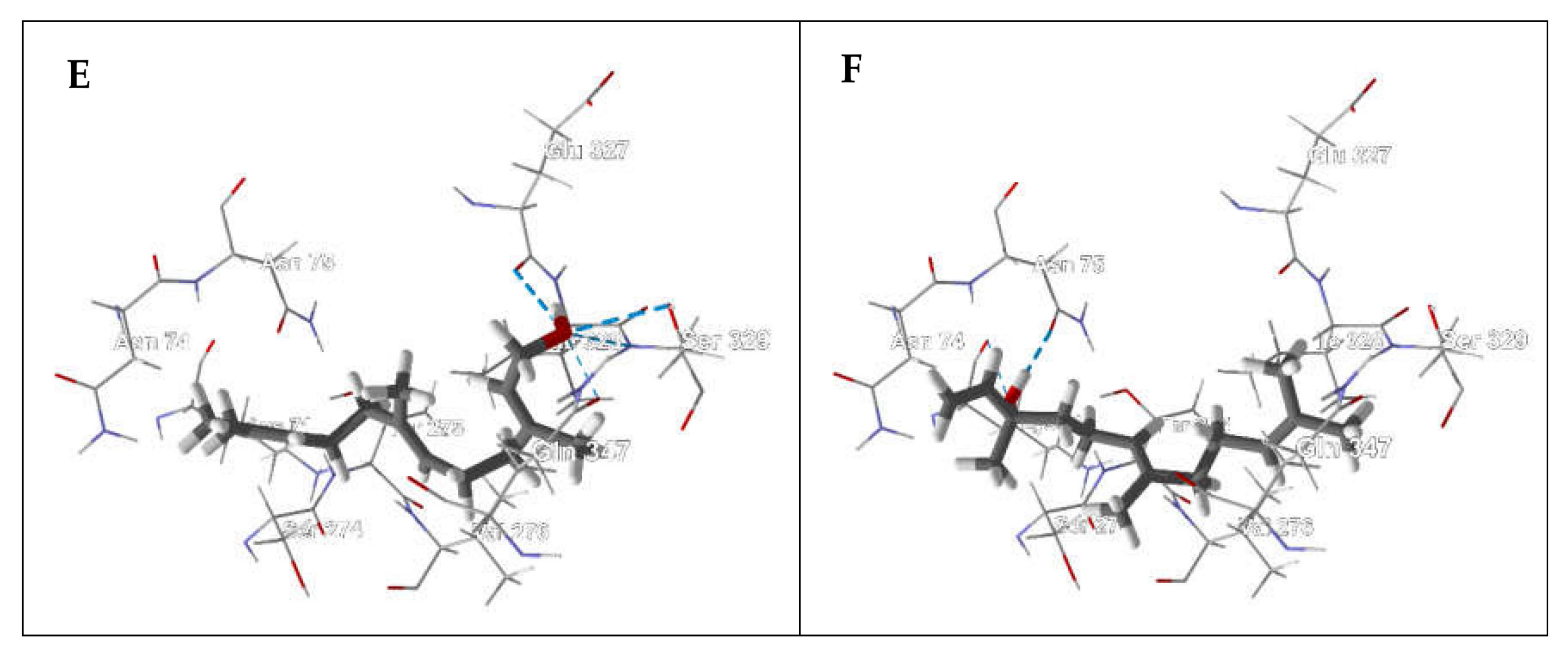
Lowest-energy docked poses of (*E*,*E*)-α-farnesene, (*E*)-β-farnesene, (*E*,*E*)-farnesol, and (*E*)-nerolidol with SARS-CoV-2 endoribonuclease (PDB: 6VWW). (**A**) Ribbon structure of the enzyme and (*E*,*E*)-α-farnesene (green), (*E*)-β-farnesene (aqua), (*E*,*E*)-farnesol (magenta), and (*E*)-nerolidol (orange). (**B**) Solid structure of the enzyme showing (*E*,*E*)-α-farnesene (green), (*E*)-β-farnesene (aqua), (*E*,*E*)-farnesol (magenta), and (*E*)-nerolidol (orange) in the binding cleft. (**C**) Lowest-energy docked pose of (*E*,*E*)-α-farnesene in the binding site. (**D**) Lowest-energy docked pose of (*E*)-β-farnesene in the binding site. (**E**) Lowest-energy docked pose of (*E*,*E*)-farnesol in the binding site. (**F**) Lowest-energy docked pose of (*E*)-nerolidol in the binding site. Hydrogen bonds are indicated with blue dashed lines.

Non-structural protein 15 (Nsp15) of SARS-CoV-2 is an endoribonuclease that preferentially cleaves RNA at uridylate. Furthermore, it has been shown that SARS-CoV Nsp15/NendoU is required for successful viral infection [109]. The best docking ligands for SARS-CoV Nsp15/NendoU are (*E*,*E*)-α-farnesene (DS_norm_ = −107.5 kJ/mol), (*E*)-β-farnesene (DS_norm_ = −105.0 kJ/mol), (*E*,*E*)-farnesol (DS_norm_ = −104.6 kJ/mol), and (*E*)-nerolidol (DS_norm_ = −101.6 kJ/mol). All of these sesquiterpenoids preferentially docked into a binding site formed by amino acid residues Gln347, Ile328, Val276, Ser274, Thr275, Ser329, Asn74, Asn75, Glu327, and Lys71 (Figure 3). In addition to van der Waals interactions, (*E*,*E*)-farnesol showed hydrogen-bonding interactions with Ser329 and Glu327, while (*E*)-nerolidol hydrogen bonded with Asn75 and Lys71 (Figure 3). Unfortunately, the docking scores for these ligands as well as the scores of the other essential oil components with this protein are too low for it to be considered a viable target (see Table 3).

**Figure 4:**
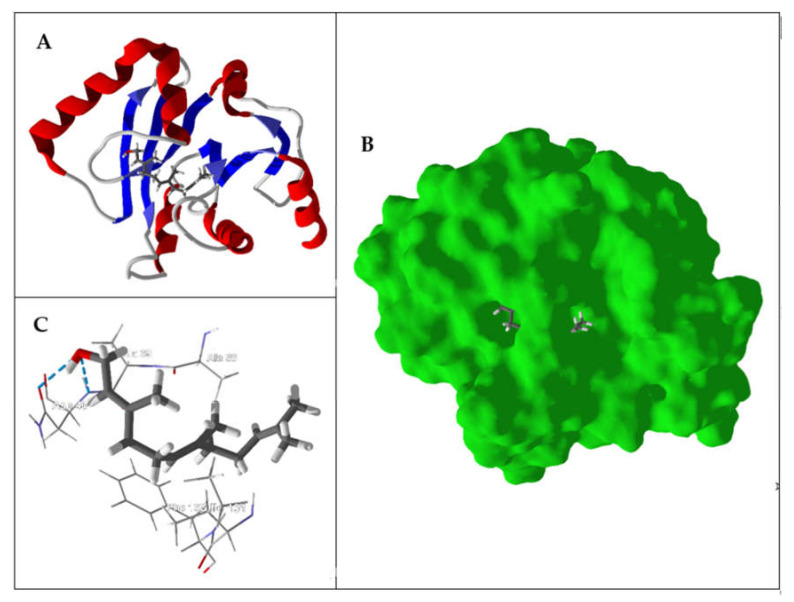
Lowest-energy docked pose of (*E*,*E*)-farnesol with SARS-CoV-2 ADP ribose phosphatase (PDB: 6W02). (**A**) Ribbon structure of the enzyme and the docked ligand. (**B**) Solid structure of the enzyme showing (*E*,*E*)-farnesol in the binding cleft. (**C**) Amino acid residues in proximity to the docked (*E*,*E*)-farnesol (hydrogen bonds are indicated with blue dashed lines).

**Figure 5:**
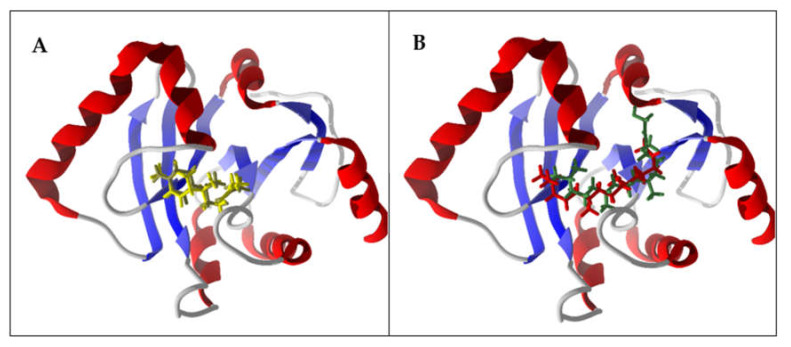
Lowest-energy docked poses of β-sesquiphellandrene, α-zingiberene, phytol, and phytone with SARS-CoV-2 ADP ribose phosphatase (PDB: 6W02). (**A**) Ribbon structure of the enzyme with β-sesquiphellandrene (brown) and α-zingiberene (yellow). (**B**) Ribbon structure of the enzyme with phytol (green) and phytone (red).

ADP ribose phosphatase (ADRP) serves to convert ADP-ribose 1″-monophosphate (Appr-1″-p) to ADP-ribose (Appr), which serves to regulate virus replication [110]. This enzyme may be dispensable in SARS-CoV-2, however [111]. Nevertheless, (*E*,*E*)-farnesol showed the most exothermic docking to SARS-CoV-2 ADRP with DS_norm_ = −121.4 kJ/mol. The binding site in SARS-CoV-2 ADRP is surrounded by Phe132, Asn40, Ile131, Ala38, and Ala39, with hydrogen-bonded interactions between the ligand alcohol and Asn40 (Figure 4). Additional essential oil components with good docking scores with SARS-CoV-2 ADRP include the sesquiterpene hydrocarbons (*E*)-β-farnesene (DS_norm_ = −116.3 kJ/mol), (*E*,*E*)-α-farnesene (DS_norm_ = −114.2 kJ/mol), β-sesquiphellandrene (DS_norm_ = −115.7 kJ/mol), and α-zingiberene (DS_norm_ = −115.4 kJ/mol); the diterpenoids phytol (DS_norm_ = −118.9 kJ/mol) and phytone (DS_norm_ = −116.9 kJ/mol); and the phenylpropanoid eugenyl acetate (DS_norm_ = −115.4 kJ/mol). Not surprisingly, β-sesquiphellandrene and α-zingiberene adopted the same docking orientation in the binding site of the enzyme (Figure 5A). Similarly, phytol and phytone occupy the same location in the binding site (Figure 5B).

RNA-dependent RNA polymerase catalyzes RNA replication from an RNA template and is an essential enzyme in RNA viruses. Because these enzymes are crucial in viral replication, they are viable targets in antiviral chemotherapy [112]. Molecular docking of essential oil components with SARS-CoV-2 RdRp showed only weak docking with this enzyme target (Table 3). The ligand with the best docking score was (*E*,*E*)-farnesol, with DS_norm_ = −89.6 kJ/mol.

**Figure 6:**
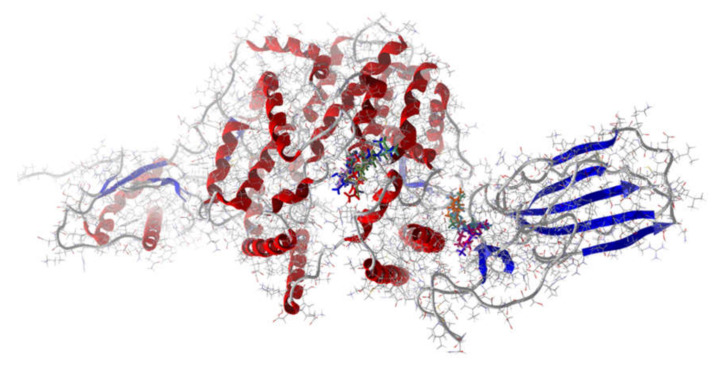
Lowest-energy docked ligands with the binding domain of SARS-CoV-2 spike protein and human angiotensin-converting enzyme 2 (ACE2) (PDB: 6M17).

The SARS-CoV-2 spike protein serves to attach to angiotensin-converting enzyme 2 (ACE2) of the human cell to be invaded. The interface between SARS-CoV-2 rS and human ACE2 would be a promising target to prevent binding of SARS-CoV-2 rS to human ACE2 [113,114]. The best docking ligands with human ACE2, i.e., normalized docking scores < -100 kJ/mol (α-bulnesene, eremanthin, (*E*,*E*)-α-farnesene, (*E*)-β-farnesene, (*E*,*E*)-farnesol, (*E*)-nerolidol, β-sesquiphellandrene, and (*Z*)-spiroether), all show docking preference to a cavity removed from the interaction interface between the SARS-CoV-2 spike protein and ACE2 (Figure 6). This cavity is a pocket surrounded by residues Pro565, Leu95, Val209, Asn210, Leu91, Lys94, Glu208, and Glu564. Because of the remote location of docking with ACE2, it is predicted that interaction of essential oil components with ACE2 will not prevent protein–protein interaction between the SARS-CoV-2 spike protein and human ACE2.

On the other hand, the lowest energy poses of essential oil components showing the strongest docking (<−80 kJ/mol; (*E*)-cinnamyl acetate, eremanthin, (*E*,*E*)-α-farnesene, (*E*)-β-farnesene, (*E*,*E*)-farnesol, and geranyl formate) with the binding domain of the SARS-CoV-2 spike protein do lie at the interface between the SARS-CoV-2 spike protein and human ACE2 (Figure 6). This docking site is a hydrophobic pocket formed by Tyr505, Tyr495, Asn501, Arg403, Tyr453, and Gly502. Unfortunately, the docking energies at this site are too weak and are unlikely, therefore, to disrupt binding between SARS-CoV-2 rS and human ACE2.

In order to compare docking scores of the essential oil components with other proteins, docking was also carried out with six randomly selected non-virus proteins: Bovine odorant binding protein (BtOBP, PDB: 1GT3), cruzain (PDB: 1ME3), torpedo acetylcholinesterase (TcAChE, PDB: 6G1U), *Bacillus anthracis* nicotinate mononucleotide adenylytransferase (BaNadD, PDB: 3HFJ), Russell’s viper phospholipase A_2_ (DrPLA2, PDB: 1FV0), and *Escherichia coli* l-aspartate aminotransferase (EcAspTA, PDB: 2Q7W). Docking scores for these proteins are summarized in Table 4.

The docking results of the essential oil components with the six randomly selected proteins indicate the best docking ligands to SARS-CoV-2 targets (i.e., (*E*,*E*)-α-farnesene, (*E*)-β-farnesene, and (*E*,*E*)-farnesol) have better docking energies with other proteins. These three sesquiterpenes have docking energies of −129.8, −122.7, and −133.0 kJ/mol with TcAChE, respectively, and −131.8, −131.8, and −135.6 kJ/mol, respectively, with BaNadD. Indeed, most of the essential oil ligands have better docking properties with one or more of the random proteins compared to the SARS-CoV-2 proteins.

Based on the docking energies of essential oil components with key protein targets of SARS-CoV-2, the individual essential oil components cannot be considered viable chemotherapeutic agents for interaction with the SARS-CoV-2 target proteins. However, essential oils are complex mixtures of compounds and several essential oil components may act synergistically to inhibit the virus. Astani and co-workers have shown, for example, that the antiviral activity (HSV-1) of *Eucalyptus* oil is much greater than the major component 1,8-cineole, and that tea tree oil has a greater antiviral activity than its components terpinen-4-ol, γ-terpinene, and α-terpinene [52].

Synergistic effects have also been observed between essential oils and synthetic antiviral agents. Civitelli and co-workers observed an antiviral synergism between *Mentha suaveolens* essential oil and acyclovir on HSV-1 [64]. Likewise, *Melissa officinalis* essential oil potentiated the activity of oseltamivir against avian influenza virus H9N2 [115]. Furthermore, essential oils are lipophilic and therefore may also serve to disintegrate viral membranes [116].

Outside of antiviral activity, there may be some relief of symptoms of COVID-19 provided by essential oils. For example, linalool [117,118], β-caryophyllene [119,120], and 1,8-cineole [121,122] have both anti-inflammatory and antinociceptive activity; menthol [123,124], camphor [125,126], and thymol [127] have antitussive activities.

## 3. Materials and Methods

### 3.1. Bibliographic Search Criteria

The bibliographic research was performed using the databases Google Scholar, Pubmed, Science Direct, Medline, and Scopus. The keywords applied were “antiviral activity” and “essential oils”, “antiviral activity” and “volatile compounds”, and “essential oils” and “respiratory diseases”.

### 3.2. Ligand Selection

The major components (>5%) of essential oils and pure essential oil components that have been screened against human pathogenic viruses were selected. In the case where enantiomers are known to be natural products, both structures were selected. A total of 171 essential oil components were used in the virtual screening.

### 3.3. Molecular Docking

Each ligand structure was prepared using Spartan ’18 v. 1.4.4 (Wavefunction, Inc., Irvine, CA, USA). The lowest-energy conformations of the ligands were determined and used as starting structures in the molecular docking. This is particularly important to include all potential conformations in medium-sized rings where interconversion between conformations may be hindered (e.g., bicyclogermacrene, costunolide, curdione, germacrene D, germacrone, and α-humulene). A total of six protein targets of SARS-CoV-2 from the Protein Data Bank (PDB), represented by a total of 17 structures, were used in the molecular docking, including SARS-CoV-2 main protease (PDB: 5R7Z, 5R80, 5R81, 5R82, 5R83, 5R84, 6LU7, 6M03, and 6Y84), SARS-CoV-2 endoribonuclease (PDB: 6VWW), SARS-CoV-2 ADP ribose phosphatase (PDB: 6W01 and 6W02), SARS-CoV-2 RNA-dependent RNA polymerase (PDB: 6M71), SARS-CoV-2 spike protein binding domain (PDB: 6M0J, 6VX1, 6VW1, and 6M17), and the human angiotensin-converting enzyme (PDB: 6M0J, 6VX1, 6VW1, and 6M17). Molecular docking was carried out using Molegro Virtual Docker v. 6.0.1 (Aarhus, Denmark) as previously reported [128,129]. Briefly, a 15-Å radius sphere centered on the binding sites of each protein structure in order to permit each ligand to search. In the case of the spike protein and human ACE2, the docking sphere was located at the interface between the spike protein and ACE2. In one case, ACE2 was removed and docking was carried out with the spike protein, and in the other case, the spike protein was removed and docking was carried out with ACE2. Standard protonation states of each protein, based on neutral pH, were used, and charges were assigned based on standard templates as part of the Molegro Virtual Docker program. Each protein was used as a rigid model without protein relaxation. Flexible-ligand models were used in the docking optimizations. Different orientations of the ligands were searched and ranked based on their “rerank” energy scores. A minimum of 100 runs for each ligand was carried out. In analyzing the docking scores, we accounted for the recognized bias due to molecular weight [130,131,132] using the scheme: DS_norm_ = 7.2 × E_dock_/MW^⅓^, where DS_norm_ is the normalized docking score, *E*_dock_ is the MolDock re-rank score, MW is the molecular weight, and 7.2 is a scaling constant to ensure the average DS_norm_ values are comparable to those of E_dock_ [128]. The best docking results are summarized in Table 1.

## 4. Conclusions

A molecular docking analysis was carried out using 171 essential oil components with the SARS-CoV-2 main protease (SARS-CoV-2 M^pro^), SARS-CoV-2 endoribonucleoase (SARS-CoV-2 Nsp15/NendoU), SARS-CoV-2 ADP-ribose-1″-phosphatase (SARS-CoV-2 ADRP), SARS-CoV-2 RNA-dependent RNA polymerase (SARS-CoV-2 RdRp), the binding domain of the SARS-CoV-2 spike protein (SARS-CoV-2 rS), and human angiotensin-converting enzyme (hACE2). The best docking ligands for the SARS-CoV target proteins were (*E*,*E*)-α-farnesene, (*E*)-β-farnesene, and (*E*,*E*)-farnesol. The docking energies were relatively weak, however, and are unlikely to interact with the virus targets. However, essential oil components may act synergistically, essential oils may potentiate other antiviral agents, or they may provide some relief of COVID-19 symptoms.

## Figures and Tables

**Table 1:** Essential oils showing antiviral activity.

Essential Oil	Major Components	Assay	IC_50_ (μg/mL)	Reference
*Aloysia gratissima* (Gillies & Hook.) Tronc.	caryophyllene oxide (15.8%), guaiol (17.4%) ^a^, chrysanthenyl acetate (5.6%), and limonene oxide (5.3%)	Plaque reduction assay (Vero cells), HSV-1	65.0	[37]
*Aloysia triphylla* Royle	α-thujone (22.9%), *cis*−carveol (17.5%), carvone (13.2%), and limonene (12.7%)	Plaque reduction assay (Vero cells), HSV-1	>250	[38]
*Artemisia arborescens* L.	camphor (35.7%), β-thujone (24.0%), and chamazulene (7.7%)	Plaque reduction assay (Vero cells), HSV-1	25% inhibition at 100 μg/mL	[39]
*Artemisia arborescens* L.	*Artemisia arborescens* L.	β-thujone (45.0%), camphor (6.8%), and chamazulene (22.7%) ^c^	Plaque reduction assay (Vero cells), HSV-1, HSV-2	2.4, 4.1	[40]
*Artemisia douglasiana* Besser	α-thujone (68.3%) and β-thujone (7.5%)	Plaque reduction assay (Vero cells), HSV-1	83	[37]
*Artemisia kermanensis* Podlech (syn. *Seriphidium kermanense* (Podlech) K. Bremer & Humphries	α-thujone (13.8%), camphor (10.2%), and β-thujone (6.2%)	Plaque reduction assay (Vero cells), HSV-1	40	[41]
*Artemisia mendozana* DC.	camphor (22.4%), artemiseole (11.7%), artemisia alcohol (10.8%), and borneol (7.2%)	Plaque reduction assay, DENV−2, JUNV and HSV-1	129.3, 178.6, and 153.7	[38]
*Artemisia princeps* Pamp.	borneol (12.1%), α-thujone (8.7%), τ−cadinol, (6.7%), and 1,8-cineole (6.2%)	Plaque reduction assay, murine norovirus−1	64% inhibition at 0.01%	[42]
		Plaque reduction assay, feline calicivirus−F9	48% inhibition at 0.1%	
*Artemisia vulgaris* L.	α-thujone (38.1%), β-thujone (10.6%), and 1,8-cineole (8.8%)	Virus yield assay (Vero cells), yellow fever virus (YFV)	100 μg/mL (100% inhibition)	[43]
*Ayapana triplinervis* (Vahl) R.M. King & H. Rob.	thymohydroquinone dimethyl ether	Plaque reduction assay (A549 cells), Zika virus	38.0	[44]
*Buddleja cordobensis* Griseb.	caryophyllene oxide (32.1%), β-caryophyllene (16.5%), and α-copaene (8.5%)	Plaque reduction assay, DENV−2, JUNV and HSV-1	86.4, 39.0, and 54.1	[38]
*Cedrus libani* A. Rich.	himachalol (22.5%), β-himachalene (21.9%), and α-himachalene (10.5%)	Cytopathic effect (CPE) on Vero cells, HSV-1	440	[45]
*Cinnamomum zeylanicum* Blume (syn. *Cinnamomum verum* J. Presl)	eugenol (75–85%), linalool (1.6−8.5%), (*E*)−cinnamaldehyde (0.6−1.5%), (*E*)−cinnamyl acetate (0.7–2.6%), β-caryophyllene (0.5–6.7%), eugenyl acetate (0.1–2.9%), and benzyl benzoate (0.1–8.3%)	Influenza type A (H1N1)		[46]
*Cinnamomum zeylanicum* Blume (syn. *Cinnamomum verum* J. Presl)	(*E*)−cinnamaldehyde (63.9%), eugenol (7.0%), and (*E*)−cinnamyl acetate (5.1%) ^c^	Plaque reduction assay (HeLa cells), HSV-2	82	[47]
*Citrus* × *bergamia* Risso & Poit.	limonene (23–55%), linalool (2–37%), linalyl acetate (12–41%), β-pinene (up to 10%), and γ-terpinene (up to 10%)	Influenza virus type A H1N1	100% inhibition at 0.3%	[46]
*Citrus limonum* Risso	limonene (54.6%), γ-terpinene (19.1%), and β-pinene (14.5%) ^d^	Plaque reduction assay (Vero cells), HSV-1	2500	[48]
*Citrus reshni* Hort ex Tan. (leaf EO)	sabinene (40.5%), linalool (23.3%), and terpinen-4-ol (8.3%)	Plaque reduction assay, influenza A virus H5N1	19.4% inhibition at 0.1 μL/mL	[36]
*Citrus reshni* Hort ex Tan. (unripe fruit peel EO)	limonene (82.4%) and linalool (7.2%)	Plaque reduction assay, influenza A virus H5N1	61.5% inhibition at 1.5 μL/mL	[36]
*Citrus reshni* Hort ex Tan. (ripe fruit peel EO)	limonene (91.6%)	Plaque reduction assay, influenza A virus H5N1	50% inhibition at 1.5 μL/mL	[36]
*Cupressus sempervirens* L.	α-pinene (51.9%) and δ-3-carene (24.9%) ^c^	Plaque reduction assay (Vero cells), HSV-1	6600	[48]
*Cymbopogon citratus* (DC.) Stapf	geranial (40.2%), neral (30.6%), and geraniol (6.7%) ^c^	Plaque reduction assay (Vero cells), HSV-1	91	[48]
*Cymbopogon flexuosus* (Nees) Will. Watson	geranial (48–54%) and neral (29–33%)	Influenza virus type A (H1N1)		[46]
*Cynanchum stauntonii* (Decne.) Schltr. ex H. Lév.	(2*E*,4*E*)-decadienal (23.0%), γ-nonalactone (4.2%), 5-pentyl-2(3H)−furanone (3.8%), and 3-isopropyl-1-pentanol (3.5%)	Influenza type A (H1N1)	64	[46]
*Dysphania ambrosioides* (L.) Mosyakin & Clemants	*cis*-ascaridole (60.7%) and *m*-cymene (22.2%)	Plaque reduction assay, Coxsackie virus B4	21.75	[49]
*Eucalyptus astringens* (Maiden) Maiden	1,8-cineole (42.0%), α-pinene (22.0%), and trans−pinocarveol (7.0%)	Vero cells, Coxsakie virus B3	13.0	[50]
*Eucalyptus bicostata* Maiden, Blakely & Simmonds	1,8-cineole (68.0%), globulol (5.4%), and *trans*−pinocarveol (4.6%)	Vero cells, Coxsakie virus B3	13.6	[50]
*Eucalyptus caesia* Benth.	1,8-cineole (40.2%), *p*-cymene (14.1%), γ-terpinene (12.4%), α-pinene (7.7%), and terpinen-4-ol (5.6%)	Plaque reduction assay (Vero cells), HSV-1	70	[41]
*Eucalyptus camaldulensis* Dehnh.	α-terpinene (26.3%), α-terpineol (9.1%), and camphene (8.5%) ^e^	Plaque reduction assay, Rotavirus Wa strain	50% inhibition at 10% EO concentration	[51]
*Eucalyptus camaldulensis* Dehnh.	α-terpinene (26.3%), α-terpineol (9.1%), and camphene (8.5%) ^e^	Plaque reduction assay, Coxsackie virus B4	53.3% at 10% EO concentration	[51]
*Eucalyptus camaldulensis* Dehnh.	α-terpinene (26.3%), α-terpineol (9.1%), and camphene (8.5%) ^e^	Plaque reduction assay, HSV-1	90% at 10% EO concentration	[51]
*Eucalyptus camaldulensis* Dehnh.	α-terpinene (26.3%), α-terpineol (9.1%), and camphene (8.5%) ^e^	Plaque reduction assay, adenovirus type 7	0% at 10% EO concentration	[51]
*Eucalyptus cinereal* F. Muell. ex Benth.	1,8-cineole (70.4%), α-pinene (4.5%), and limonene (3.7%)	Vero cells, Coxsakie virus B3	13.0	[50]
*Eucalyptus globulus* Labill.	1,8-cineole (63.8%) and α-pinene (14.0%) ^f^	Plaque reduction assay (Vero cells), HSV-1	1700	[48]
*Eucalyptus globulus* Labill.	1,8-cineole (63.8%) and α-pinene (14.0%) ^f^	Plaque reduction assay (RC-37 cells), HSV-1	55.0	[52]
*Eucalyptus lehmannii* (Schauer) Benth.	1,8-cineole (59.6%), α-pinene (17.6%), and α-terpineol (8.7%)	Vero cells, Coxsakie virus B3	11.5	[50]
*Eucalyptus leucoxylon* F. Muell.	1,8-cineole (59.2%), α-pinene (7.8%), and α-terpineol (4.3%)	Vero cells, Coxsakie virus B3	8.1	[50]
*Eucalyptus maidenii* F. Muell.	1,8-cineole (57.8%), *p*-cymene (7.4%), and α-pinene (7.3%)	Vero cells, Coxsakie virus B3	14.5	[50]
*Eucalyptus odorata* Behr	cryptone (20.9%), *p*-cymene (16.7%), phellandral (6.6%), and cuminal (6.6%)	Vero cells, Coxsakie virus B3	19.2	[50]
*Eucalyptus sideroxylon* A. Cunn. ex Woolls	1,8-cineole (69.2%), α-pinene (6.9%), and α-terpineol (5.4%)	Vero cells, Coxsakie virus B3	12.3	[50]
*Eupatorium patens* D. Don ex Hook. & Arn.	germacrene D (36.2%), β-caryophyllene (14.1%), and bicyclogermacrene (7.0%)	Plaque reduction assay (Vero cells), HSV-1	125	[37]
*Fortunella margarita* (Lour.) Swingle (leaf EO)	α-terpineol (55.5%), carvone (5.7%), carveol (5.5%), γ-muurolene (5.5%), and citronellal (5.0%)	MTT assay, reasserted avian influenza A virus, H5N1	6.77	[53]
*Fortunella margarita* (Lour.) Swingle (fruit EO)	β-eudesmol (28.3%), α-muurolene (10.3%), β-gurjunene (10.0%), γ-eudesmol (8.4%), and γ-muurolene (6.6%)	MTT assay, reasserted avian influenza A virus, H5N1	38.89	[53]
*Gaillardia megapotamica* (Spreng.) Baker	β-pinene (24.2%), (*Z*)-β-ocimene (16.5%), α-pinene (7.7%), limonene (7.5%), and β-caryophyllene (6.7%)	Plaque reduction assay, DENV−2, JUNV and HSV-1	140.6, 49.8, and 99.1	[38]
*Heterothalamus alienus* (Spreng.) Kuntze	β-pinene (35.5%), spathulenol (10.7%), and germacrene D (6.8%)	Plaque reduction assay DENV−2, JUNV and HSV-1	122.3, 44.2 and 148.4	[38]
*Heterotheca latifolia* Buckley	borneol (40.0%), camphor (24.3%), and limonene (5.1%)	Plaque reduction assay (Vero cells), HSV-1	>150	[37]
*Houttuynia cordata* Thunb.	decanal (3.4−8.9%), decanol (up to 7.0%), 2−undecanone (23.0−36.1%), decanoic acid (1.4−6.3%), dodecanal (up to 7.3%), and 2−tridecanone (2.6−5.6%)	Influenza type A (H1N1)	48	[54]
*Hyptis mutabilis* (Rich.) Briq.	fenchone (17.1%), 1,8-cineole (12.6%), β-caryophyllene (10.9%), bicyclogermacrene (8.7%), and germacrene D (6.2%)	Plaque reduction assay (Vero cells), HSV-2 (Human Herpesvirus type 1)	79.01	[55]
*Hyptis mutabilis* (Rich.) Briq.	germacrene D (15.1%), β-caryophyllene (13.3%), curzerene (13.3%), and bicyclogermacrene (13.2%)	Plaque reduction assay (Vero cells)	>150	[37]
*Hyssopus officinalis* L.	*cis*−pinocamphone (40.1%), *trans*−pinocamphone (13.3%), β-pinene (10.7%), and β-phellandrene (5.3%) ^c^	Plaque reduction assay (RC-37 cells), HSV-1	1	[56]
*Hyssopus officinalis* L.	*cis*−pinocamphone (40.1%), *trans*−pinocamphone (13.3%), β-pinene (10.7%), and β-phellandrene (5.3%) ^c^	Plaque reduction assay (RC-37 cells), HSV-2	6	[57]
*Illicium verum* Hook. f.	(*E*)−anethole (90.5%) ^c^	Plaque reduction assay (RC-37 cells), HSV-1	40	[58]
*Illicium verum* Hook. f.	(*E*)−anethole (90.5%) ^c^	Plaque reduction assay (RC-37 cells), HSV-1	1	[59]
*Illicium verum* Hook. f.	(*E*)−anethole (90.5%) ^c^	Plaque reduction assay (RC-37 cells), HSV-2	30	[57]
*Jungia polita* Griseb.	caryophyllene oxide (9.2%) and β-caryophyllene (8.1%)	Plaque reduction assay DENV−2, JUNV and HSV-1	39.8, 134.2 and 136.4	[38]
*Juniperus communis* L.	α-pinene (46.7%), myrcene (15.0%), sabinene (13.2%), and limonene (7.0%) ^c^	Plaque reduction assay (Vero cells), HSV-1	>10000	[48]
*Lavandula angustifolia* Mill.	linalyl acetate (37.0−43.6%), linalool (19.7−39.1%), geraniol (up to 9.3%), β-caryophyllene (up to 5.1%), terpinene−4−ol (up to 14.9%), lavandulol (up to 1.5%), lavandulyl acetate (up to 5.5%), 1,8-cineole (up to 4.1%), and borneol (up to 6.4%)	85% *in vitro* inhibition of influenza type A (H1N1)		[46]
*Lavandula latifolia* Medik.	linalool (31.9%), 1,8-cineole (18.8%), and borneol (10.1%) ^g^	Plaque reduction assay (Vero cells), HSV-1	2200	[48]
*Lepechinia salviifolia* (Kunth) Epling	camphor (10.3%), limonene (9.7%), *p*−mentha−1(7),8−diene (7.4%), α-pinene (6.9%), and γ-terpinene (6.7%)	Plaque reduction assay (Vero cells), HSV-1, HSV-2	68.8, 81.9	[55]
*Lepechinia vulcanicola* J.R.I. Wood	limonene (18.9%), germacrene D (10.4%), 1−octen−3−ol (8.8%), β-caryophyllene (8.7%), and α-pinene (8.2%)	Plaque reduction assay (Vero cells), HSV-1, HSV-2	112, 68.9	[55]
*Leptospermum scoparium* J.R. Forst. & G. Forst.	calamene (16.0%), leptospermone (14.5%), δ−cadinene (6.1%), flavesone (4.5%), viridiflorene (4.4%), and isoleptospermone (3.9%)	Plaque reduction assay (RC-37 cells), HSV-1, HSV-2	0.96, 0.58	[60]
*Lippia alba* (Mill.) N.E. Br. ex Britton & P. Wilson	carvone (51.0%), Limonene (33%), and bicyclosesquiphellandrene (7.0%)	Virus yield assay (Vero cells), yellow fever virus (YFV)	100 μg/mL (100% inhibition)	[43]
*Lippia alba* (Mill.) N.E. Br. ex Britton & P. Wilson	carvone (39.7%), limonene (30.6%), and bicyclosesquiphellandrene (8.9%)	Plaque reduction assay (Vero cells), DENV−1, DENV−2, DENV−3, DENV−4, YFV 17 DD	10.1, 0.4, 32.6, 21.1, 4.9	[61]
*Lippia alba* (Mill.) N.E. Br. ex Britton & P. Wilson	carvone (39.7%), limonene (30.6%), and bicyclosesquiphellandrene (8.9%)	Plaque reduction assay (Vero cells), Yellow fever (YFV)	4.3	[62]
*Lippia citriodora* Kunth (syn. *Aloysia citriodora* Palau)	geranial (18.9%), neral (15.6%), limonene (10.7%), and 1,8-cineole (5.0)	Plaque reduction assay (Vero cells), DENV−1, DENV−2, DENV−3, DENV−4, YFV 17 DD	1.9, 2.9, 2.6, 33.7, 5.7	[61]
*Lippia citriodora* Kunth (syn. *Aloysia citriodora* Palau)	geranial (18.9%), neral (15.6%), limonene (10.7%), and 1,8-cineole (5.0)	Plaque reduction assay (Vero cells), Yellow fever (YFV)	19.4	[62]
*Lippia graveolens* Kunth	carvacrol (56.8%), *o*−cymene (32.1%), and γ-terpinene (3.7%) ^h^	MTT assay (Mardin−Darby bovine kidney cells), HSV-1, ACVR−HSV-1 (acyclovir−resistant HSV-1), HRSV (human respiratory syncytial virus)	99.6, 55.9, 68.0	[63]
*Lippia junelliana* (Moldenke) Tronc.	piperitenone oxide (= rotundifolone) (36.5%), limonene (23.1%), camphor (7.9%), and spathulenol (6.5%)	Plaque reduction assay (Vero cells), HSV-1	>150	[37]
*Lippia origanoides*	carvacrol (44.0%), thymol (15.0%), and γ-terpinene (10.0%)	Virus yield assay (Vero cells), yellow fever virus (YFV)	11.1 μg/mL (100% inhibition)	[43]
*Lippia turbinata* Griseb.	limonene (60.6%), piperitenone oxide (17.4%), and β-caryophyllene (6.4%)	Plaque reduction assay (Vero cells), HSV-1	> 150	[37]
*Matricaria recutita* L.	α-bisabolol oxide A (13.4–55.9%), α-bisabolol oxide B (8.4–25.1%), bisabolone oxide A (2.9–11.4%), *cis*−bicycloether (= (*Z*)−spiroether) (3.6–17.7%), and (*E*)-β-farnesene (1.9–10.4%) ^i^	Plaque reduction assay (RC-37 cells), HSV-1	0.3	[58]
*Matricaria recutita* L.	α-bisabolol oxide A (13.4–55.9%), α-bisabolol oxide B (8.4–25.1%), bisabolone oxide A (2.9−11.4%), *cis*−bicycloether (= (*Z*)−spiroether) (3.6–17.7%), and (*E*)-β-farnesene (1.9–10.4%) ^i^	Plaque reduction assay (RC-37 cells), HSV-2	1.5	[57]
*Melaleuca alternifolia* Cheel	terpinen-4-ol (36.71%), γ-terpinene (22.20%), and α-terpinene (10.10%)	Plaque reduction assay, influenza A⁄PR ⁄ 8 virus subtype H1N1	60	[31]
*Melaleuca alternifolia* Cheel	terpinen-4-ol (30–48%), γ-terpinene (10–28%), α-terpinene (5–13%), 1,8-cineole (up to 15%), terpinolene (1.5−5%), *p*-cymene (0.5–12%), α-pinene (1–6%), and α-terpineol (1.5–8%)	100% inhibition of influenza type A (H1N1) virus at 0.01%; type A (H11N9) virus to tea tree oil vapor caused 100% inhibition		[46]
*Melaleuca alternifolia* Cheel	terpinen-4-ol (47.5%), γ-terpinene (20.2%), and α-terpinene (8.6%) c	Plaque reduction assay (Vero cells), HSV-1	2700	[48]
*Melaleuca alternifolia* Cheel	terpinen-4-ol (47.5%), γ-terpinene (20.2%), and α-terpinene (8.6%) c	Plaque reduction assay (Vero cells), HSV-1	13.2	[64]
*Melaleuca alternifolia* Cheel	terpinen-4-ol (47.5%), γ-terpinene (20.2%), and α-terpinene (8.6%) c	Plaque reduction assay (RC-37 cells), HSV-1	2	[52]
*Melaleuca alternifolia* Cheel	terpinen-4-ol (47.5%), γ-terpinene (20.2%), and α-terpinene (8.6%) c	Plaque reduction assay (HeLa cells), HSV-2	2700	[47]
*Melaleuca alternifolia* Cheel	terpinen-4-ol (47.5%), γ-terpinene (20.2%), and α-terpinene (8.6%) c	Plaque reduction assay (Vero cells), HSV-1	significant plaque reduction at 10 and 5% v/v	[65]
*Melaleuca armillaris* (Sol. ex Gaertn.) Sm.	1,8-cineole (33.9%), terpinen-4-ol (18.8%), and γ-terpinene (10.4%)	Plaque reduction assay (Vero cells), HSV-1	99% plaque reduction (concentration not given)	[66]
*Melaleuca ericifolia* Sm.	methyl eugenol (96.84%)	Plaque reduction assay (Vero cells), HSV-1	91.5% plaque reduction (concentration not given)	[66]
*Melaleuca leucadendra* (L.) L.	1,8-cineole (64.3%), α-terpineol (11.0%), and valencene (3.91%)	Plaque reduction assay (Vero cells), HSV-1	92% plaque reduction (concentration not given)	[66]
*Melissa officinalis* L.	neral (17−32%), geranial (23–43%), linalool (up to 9.0%), citronellal (0.7–20.3%), geraniol (up to 23.2%), β-caryophyllene (up to 11.3%), and caryophyllene oxide (0.4−31.7%)	influenza A virus (H9N2)	Significant reduction at 5 μg/mL	[46]
*Melissa officinalis* L.	β-cubebene (15.4%), β-caryophyllene (14.2%), α-cadinol (7.2%), geranial (6.6%), and neral (5.8%)	Plaque reduction assay (HEp−2 cells), HSV-2	21	[67]
*Melissa officinalis* L.	geranial (20.1%), β-caryophyllene (17.3%), and neral (13.6%)	Plaque reduction assay (RC-37 cells), HSV-1, HSV-2	4, 0.8	[68]
*Mentha* × *piperita* L.	menthol (42.8%), menthone (14.6%), and isomenthone (5.9%)	Plaque reduction assay (RC-37 cells), HSV-1, HSV-2	20, 8	[69]
*Mentha* × *piperita* L.	menthol (43.8%), menthone (19.7%), menthyl acetate (6.5%), and 1,8-cineole (5.0%) c	Plaque reduction assay (Vero cells), HSV-1	2200	[48]
*Mentha suaveolens* Ehrh.	limonene (7.4%), isopulegol (12.0%), and piperitenone oxide (41.8%)	Plaque reduction assay, cytopathogenic murine norovirus	0.87	[70]
*Mentha suaveolens* Ehrh.	piperitenone oxide (86.9%)	Plaque reduction assay (Vero cells), HSV-1	5.1	[64]
*Minthostachys mollis* Griseb.	*cis*−piperitone epoxide (29.9%), piperitenone oxide (25.6%), menthone (7.4%), germacrene D (5.8%), and pulegone (5.5%)	Plaque reduction assay (Vero cells), HSV-1, HSV-2	70.7, 68.0	[55]
*Ocimum basilicum* var. *album* (L.) Benth.	linalool (53.8%) and eugenol (12.6%) ^j^	Plaque reduction assay (Vero cells), HSV-1	>10000	[48]
*Ocimum campechianum* Mill.	methyl eugenol (53.9%), β-caryophyllene (13.0%), α-bulnesene (5.4%), germacrene D (3.4%), and α-humulene (3.3%)	Plaque reduction assay (Vero cells), HSV-2	74.33	[55]
*Origanum elongatum* Emb. (leaf EO)	*p*-cymene (16.2%), γ-terpinene (13.5%), thymol (14.2%), and carvacrol (19.2%)	Plaque reduction assay, cytopathogenic murine norovirus	0.37	[70]
*Origanum elongatum* Emb. (inflorescence EO)	*p*-cymene (16.1%), γ-terpinene (7.3%), and carvacrol (40.1%),	Plaque reduction assay, cytopathogenic murine norovirus	0.75	[70]
*Origanum majorana* L.	terpinen-4-ol (28.9%), γ-terpinene (14.9%), *trans*−sabinene hydrate (9.5%), α-terpinene (8.7%), and sabinene (7.2%) ^c^	Plaque reduction assay (Vero cells), HSV-1	2800	[48]
*Origanum majorana* L.	terpinen-4-ol (28.9%), γ-terpinene (14.9%), *trans*−sabinene hydrate (9.5%), α-terpinene (8.7%), and sabinene (7.2%) ^c^	Plaque reduction assay (HeLa cells), HSV-2	520	[47]
*Origanum vulgare* L.	trans−sabinene hydrate (21.0%), thymol (11.0%), and carvacrol methyl ether (11.0%)	Virus yield assay (Vero cells), yellow fever virus (YFV)	100 μg/mL (100% inhibition)	[43]
*Osmunda regalis* L.	hexahydrofarnesyl acetone ( = phytone) (11.8%), 2,4−di−*t*−butylphenol (6.8%), phytol (6.5%), hexadecene (4.1%), and octadecene (4.4%)	Plaque reduction assay, Coxsackie virus B4 type 2	2.24	[71]
*Pectis odorata* Griseb.	limonene (50.2%), neral (27.2%), and geranial (23.6%)	Plaque reduction assay DENV−2, JUNV and HSV-1	39.6, 36.6, and 71.5	[38]
*Pelargonium graveolens* L’Hér. ex Aiton	citronellol (21.9–37.5%), citronellyl formate (9.8–20.6%), geraniol (6.0–16.5%), geranyl formate (1.5–6.5%), menthone (up to 13%), isomenthone (up to 9.9%), and linalool (0.8−14.9%)	influenza type A (H1N1)	95% inhibition at 0.3%	[46]
*Pinus mugo* Turra	δ-3-carene (23.9%), α-pinene (17.9%), β-pinene (7.8%), and β-phellandrene (7.2%) ^k^	Plaque reduction assay (RC-37 cells), HSV-1	7	[58]
*Ravensara aromatica* Sonn. (syn. *Cryptocarya agathophylla* van der Werff)	1,8-cineole (52.6%), α-terpineol (12.4%), and sabinene (11.0%) ^l^	Plaque reduction assay (Vero cells), HSV-1	2800	[48]
*Rosmarinus officinalis* L.	α-pinene (23.9%), verbenone (15.4%), camphor (11.0%), camphene (8.7%), *p*-cymene (7.5%), and 3−octanone (5.6%)	Plaque reduction assay (Vero cells), HSV-1	60	[41]
*Rosmarinus officinalis* L.	1,8-cineole (45.9%), α-pinene (12.0%), camphor (10.9%), and β-pinene (6.3%) ^b^	Plaque reduction assay (Vero cells), HSV-1	2700	[48]
*Salvia fruticosa* Mill.	1,8-cineole (47.5%), camphor (9.0%), β-thujone (7.6%), and α-thujone (4.3%)	Plaque reduction assay (Vero cells), HSV-1 and HSV-2	1300	[72]
*Santalum album* L.	(*Z*)−α-santalol (45.2%), (*Z*)-β-santalol (25.4%), and (*Z*)−*trans*−α-bergamotol (7.8%) ^c^	Plaque reduction assay (Vero cells), HSV-1, HSV-2	22.7, 45.3	[73]
*Santalum album* L.	(*Z*)−α-santalol (45.2%), (*Z*)-β-santalol (25.4%), and (*Z*)−*trans*−α-bergamotol (7.8%) ^c^	Plaque reduction assay (RC-37 cells), HSV-1	2	[56]
*Santalum album* L.	(*Z*)−α-santalol (45.2%), (*Z*)-β-santalol (25.4%), and (*Z*)−*trans*−α-bergamotol (7.8%) ^c^	Plaque reduction assay (RC-37 cells), HSV-2	5	[57]
*Santolina insularis* (Gennari ex Fiori) Arrigoni	artemisia ketone (21.2%), *allo*−aromadendrene (12.7%), 1,8-cineole (9.0%), and camphene (8.5%) ^m^	Plaque reduction assay (Vero cells), HSV-1 and HSV-2	0.88, 0.7	[74]
*Satureja hortensis* L.	carvacrol (32.4%), γ-terpinene (32.0%), thymol (10.0%), and *p*-cymene (6.6%)	Plaque reduction assay (Vero cells), HSV-1	80	[41]
*Tessaria absinthioides* (Hook. & Arn.) DC.	caryophyllene oxide (12.2%), (*E*)-β-damascenone, γ-eudesmol (8.5%), α-gurjunene (5.8%), and terpinen-4-ol (5.4%)	Plaque reduction assay (Vero cells), HSV-1	105	[37]
*Thymus capitatus* (L.) Hoffmanns. & Link (unripe fruit EO)	carvacrol (68.6%), *p*-cymene (4.8%), γ-terpinene (3.0%), and β-caryophyllene (2.9%)	Plaque reduction assay, cytopathogenic murine norovirus	0.49	[70]
*Thymus capitatus* (L.) Hoffmanns. & Link (ripe fruit EO)	carvacrol (58.8%), *p*-cymene (5.6%), γ-terpinene (2.8%), and β-caryophyllene (2.6%)	Plaque reduction assay, cytopathogenic murine norovirus	0.50	[70]
*Thymus vulgaris* L.	thymol (31–50%), *p*-cymene (0.1–26.6%), and γ-terpinene (up to 22.8%)	100% inhibition of type A (H1N1) virus at 0.3% concentration		[46]
*Thymus vulgaris* L.	thymol (43.9%), carvacrol (14.4%), *p*-cymene (10.5%), β-caryophyllene (7.0%), and γ-terpinene (5.1%) ^c^	Plaque reduction assay (RC-37 cells), HSV-1	10	[56]
*Thymus vulgaris* L.	thymol (43.9%), carvacrol (14.4%), *p*-cymene (10.5%), β-caryophyllene (7.0%), and γ-terpinene (5.1%) ^c^	Plaque reduction assay (RC-37 cells), HSV-1	11	[52]
*Thymus vulgaris* L.	thymol (43.9%), carvacrol (14.4%), *p*-cymene (10.5%), β-caryophyllene (7.0%), and γ-terpinene (5.1%) ^c^	Plaque reduction assay (RC-37 cells), HSV-2	7	[57]
*Thymus willdenowii* Boiss.	1,8-cineole (34.62%), camphor (18.55%), α-pinene (9.46%), and camphene (5.38%)	Plaque reduction assay, Coxsackie virus	Inactive	[75]
*Trachyspermum ammi* (L.) Sprague	thymol (35–60%), α-pinene, *p*-cymene, and limonene	Plaque reduction assay, Japanese encephalitis virus	80% reduction at 500 μg/mL	[76]
*Zataria multiflora* Boiss.	thymol (47.3%), carvacrol (21.9%), *p*-cymene (8.6%), γ-terpinene (4.2%), and β-caryophyllene (3.0%)	Real time PCR (H9N2 subtype of AIV)	Reduced viral replication in trachea of broiler chickens	[77]
*Zataria multiflora* Boiss.	thymol (33.1%), carvacrol (25.9%), and *p*-cymene (11.3%)	Plaque reduction assay (Vero cells), HSV-1	30	[41]
*Zingiber officinale* Roscoe	α-zingiberene (32.1%), *ar*−curcumene (15.2%), β-sesquiphellandrene (10.9%), α-farnesene (7.2%), and α-phellandrene (4.4%)	Plaque reduction assay, Caprine alphaherpesvirus type I	not determined	[78]
*Zingiber officinale* Roscoe	α-zingiberene (26.4%), camphene (12.6%), β-sesquiphellandrene (9.2%), *ar*−curcumene (6.5%), β-phellandrene (6.2%), and β-bisabolene (5.1%) ^c^	Plaque reduction assay (RC-37 cells), HSV-1	2	[56]
*Zingiber officinale* Roscoe	α-zingiberene (26.4%), camphene (12.6%), β-sesquiphellandrene (9.2%), *ar*−curcumene (6.5%), β-phellandrene (6.2%), and β-bisabolene (5.1%) ^c^	Plaque reduction assay (RC-37 cells), HSV-2	1	[57]

^a^ Reported as cadinol, but see [79,80]. ^b^ Essential oil composition not reported; essential oil composition obtained from [81]. ^c^ Essential oil composition not reported; essential oil composition of commercial (dōTERRA International, Pleasant Grove, Utah, USA). ^d^ Essential oil composition not reported; essential oil composition obtained from [82]. ^e^ Essential oil composition not reported; essential oil composition obtained from [83]. ^f^ Essential oil composition not reported; essential oil composition obtained from [84]. ^g^ Essential oil composition not reported; essential oil composition obtained from [85]. ^h^ Essential oil composition not reported; essential oil composition obtained from [86]. ^i^ Essential oil composition not reported; essential oil composition obtained from [87]. ^j^ Essential oil composition not reported; essential oil composition obtained from [88]. ^k^ Essential oil composition not reported; essential oil composition obtained from [89]. ^l^ Essential oil composition not reported; essential oil composition obtained from [90]. ^m^ Essential oil composition not reported; essential oil composition obtained from [91].

**Table 2:** Antiviral activities of essential oil components.

Essential Oil Component	Assay	IC_50_ (μg/mL)	Reference
(*E*)−Anethole	Plaque reduction assay (RC-37 cells), HSV-1	20	[59]
Camphor	Plaque reduction assay (Vero cells), HSV-1	2600	[72]
Carvacrol	MTT assay (Mardin−Darby bovine kidney (MDBK) cells, HSV-1	48.6	[63]
β-Caryophyllene	Plaque reduction assay (RC-37 cells), HSV-1	0.25	[59]
Caryophyllene oxide	Plaque reduction assay (RC-37 cells), HSV-1	0.7	[59]
1,8-Cineole	Plaque reduction assay (Vero cells), HSV-1	1800	[72]
1,8-Cineole	Plaque reduction assay (RC-37 cells), HSV-1	1200	[52]
(*E*)-Cinnamaldehyde	Influenza type A (H1N1) virus	70% at a concentration of 0.53% after 3 h	[95]
(*E*)-Cinnamaldehyde	H1N1 in-vivo by inhalation in a mouse model	89% inhibition	[95]
Citral (Geranial + Neral)	Yellow fever (YFV), Vero cells	17.6	[62]
Citral (Geranial + Neral)	Plaque reduction assay (RC-37 cells), HSV-1	3.50	[52]
*p*-Cymene	Influenza, HSV-1, HSV-2, ECHO 9, Cox B1, Polio 1, Adeno 6	>500	[31]
*p*-Cymene	Plaque reduction assay (RC-37 cells), HSV-1	16	[52]
*p*-Cymene	Influenza type A (H1N1) virus	Inactive	[25]
Dodecanal	Influenza type A (H1N1) virus	51	[54]
β-Eudesmol	Plaque reduction assay (Vero cells), HSV-1	6	[59]
Eugenol	Plaque reduction assay (RC-37 cells), HSV-1	35	[59]
Eugenol	Plaque reduction assay (RC-37 cells), HSV-1	25.6	[96]
Farnesol	Plaque reduction assay (RC-37 cells), HSV-1	3.5	[59]
Germacrone	Influenza type A (H1N1) virus	1.22–1.55	[97]
Germacrone	Influenza type A (H3N2) virus	0.34	[97]
Germacrone	Influenza type B virus	1.38	[97]
Isoborneol	Plaque reduction assay (Vero cells), HSV-1	<1000	[98]
Nerolidol (natural)	Plaque reduction assay (RC-37 cells), HSV-1	4.2	[99]
Nerolidol (synthetic)	Plaque reduction assay (RC-37 cells), HSV-1	1.5	[99]
Octanal	Influenza type A (H1N1) virus	15	[54]
Patchouli alcohol	Influenza type A (H1N1)	89% inhibition at 10 μg/mL	[100]
Patchouli alcohol	Influenza type A (H2N2)	0.89	[101]
Patchouli alcohol	Influenza type A (H2N2), in-vivo test using a mouse model	70% survival rate at a dose of 5 mg/(kg day)	[101]
α-Pinene	Plaque reduction assay (RC-37 cells), HSV-1	4.5	[52]
Piperitenone oxide	Plaque reduction assay (Vero cells)	1.4	[64]
α-Terpinene	Influenza type A (H1N1) virus	Inactive	[25]
α-Terpinene	Influenza, HSV-1, HSV-2, ECHO 9, Cox B1, Polio 1, Adeno 4	>12	[31]
α-Terpinene	Plaque reduction assay (RC-37 cells), HSV-1	8.5	[52]
γ-Terpinene	Plaque reduction assay (RC-37 cells), HSV-1	7	[52]
γ-Terpinene	Influenza, HSV-1, HSV-2, ECHO 9, Cox B1, Polio 1, Adeno 5	>120	[31]
γ-Terpinene	influenza type A (H1N1) virus	inactive	[25]
Terpinen-4-ol	Influenza, HSV-1, HSV-2, ECHO 9, Cox B1, Polio 1, Adeno 2	25 (influenza) >50 (others)	[31]
Terpinen-4-ol	Plaque reduction assay (Vero cells), HSV-1	60	[52]
α-Terpineol	Plaque reduction assay (RC-37 cells), HSV-1	22	[52]
Terpinolene	Influenza, HSV-1, HSV-2, ECHO 9, Cox B1, Polio 1, Adeno 3	12 (influenza) >12 (others)	[31]
Thujones (α & β)	Plaque reduction assay (RC-37 cells), HSV-1	400	[72]
Thymol	Influenza type A (H1N1) virus	Active	[25]
Thymol	Plaque reduction assay (RC-37 cells), HSV-1	30	[52]
2-Undecanone	influenza type A (H1N1) virus	62	[54]

**Table 3:** Docking scores, normalized for molecular weight (DS_norm_, kJ/mol), of essential oil components with severe acute respiratory syndrome coronavirus 2 (SARS-CoV-2) molecular targets.

Compound	Main Protease	Endoribo- Nuclease	ADP Ribose Phosphatase	RNA-Dependent RNA Polymerase	Spike Protein Binding Domain	Angiotensin- Converting Enzyme ^a^
(*E*)-Anethole	−84.9	−83.0	−97.5	−74.0	−65.2	−83.8
*allo*-Aromadendrene	−86.6	−86.9	−95.8	−74.9	−66.1	−85.3
Artemiseole	−89.2	−83.4	−96.6	−73.0	−67.8	−78.0
(*R*)-Artemisia alcohol	−84.3	−78.3	−88.2	−66.8	−61.1	−74.7
(*S*)-Artemisia alcohol	−86.1	−85.4	−98.5	−69.5	−67.1	−77.4
Artemisia ketone	−91.0	−85.7	−97.9	−71.9	−66.9	−77.3
Ascaridole	−74.8	−68.2	−86.0	−65.2	−62.6	−64.4
Benzyl benzoate	−104.9	−96.5	−110.3	−82.5	−74.9	−96.4
(*Z*)-*trans*-α-Bergamotol	−98.0	−91.2	−105.9	−71.9	−63.6	−83.6
Bicyclogermacrene	−85.9	−88.0	−92.5	−75.6	−68.2	−86.1
Bicyclosesquiphellandrene	−79.4	−89.6	−86.0	−61.8	−62.5	−78.2
α-Bisabolol oxide A	−88.5	−87.6	−89.1	−71.5	−63.5	−89.7
α-Bisabolol oxide B	−96.6	−92.2	−101.0	−79.7	−75.2	−95.6
Bisabolone oxide A	−93.7	−87.9	−98.2	−73.3	−71.0	−78.7
(+)-Borneol	−77.4	−91.8	−100.1	−74.1	−70.9	−73.3
(−)-Borneol	−71.4	−71.5	−78.4	−56.7	−50.1	−73.7
(+)-*iso*-Borneol	−75.6	−75.3	−75.9	−60.8	−53.2	−71.8
(−)-*iso*-Borneol	−73.8	−69.2	−75.6	−54.8	−49.3	−72.4
α-Bulnesene	−95.2	−72.4	−78.4	−57.0	−57.8	−101.7
δ-Cadinene	−91.6	−90.2	−112.6	−75.2	−60.0	−93.4
τ-Cadinol	−92.3	−82.8	−82.5	−69.9	−69.8	−87.8
(*R*)-Calamene	−92.3	−83.5	−97.4	−69.0	−65.6	−87.3
(*S*)-Calamene	−88.4	−86.8	−95.9	−70.2	−64.7	−87.5
(+)-Camphene	−85.6	−78.9	−83.2	−64.4	−57.9	−72.2
(−)-Camphene	−77.2	−74.1	−87.1	−66.0	−59.8	−67.1
(+)-Camphor	−75.2	−73.1	−77.9	−63.9	−53.8	−69.4
(−)-Camphor	−72.5	−70.0	−75.6	−64.3	−52.8	−70.8
(+)-δ-3-Carene	−87.9	−78.6	−87.0	−65.0	−72.4	−75.8
(−)-δ-3-Carene	−83.9	−88.0	−90.4	−66.8	−67.3	−73.4
Carvacrol	−84.5	−86.6	−94.8	−74.1	−71.0	−81.2
Carvacrol methyl ether	−85.5	−82.5	−103.1	−74.6	−65.8	−83.7
(+)-*cis*−Carveol	−87.0	−81.8	−97.7	−76.5	−69.7	−80.4
(−)-*cis*−Carveol	−85.6	−85.0	−95.6	−76.8	−74.3	−81.2
(*R*)-Carvone	−87.7	−82.4	−98.2	−74.7	−69.2	−83.4
(*S*)-Carvone	−86.2	−83.2	−98.9	−73.2	−66.3	−82.2
(*E*)-Caryophyllene	−81.2	−82.2	−93.9	−73.4	−59.2	−75.1
Caryophyllene oxide	−80.6	−86.7	−97.0	−74.1	−66.5	−83.3
Cedrol	−82.3	−84.4	−80.1	−69.7	−58.3	−69.9
*epi*-Cedrol	−76.4	−88.4	−92.2	−68.5	−55.5	−75.9
Chamazulene	−97.6	−96.4	−110.9	−76.9	−73.7	−95.6
(−)-*cis*-Chrystanthyl acetate	−83.2	−77.5	−87.1	−71.5	−60.2	−80.9
(−)-*trans*−Chrysanthenyl acetate	−77.0	−81.4	−85.3	−65.9	−68.9	−72.4
1,8-Cineole	−72.7	−67.6	−71.5	−58.1	−58.6	−61.0
(*E*)-Cinnamaldehyde	−93.1	−85.8	−100.0	−76.4	−73.1	−81.0
(*E*)-Cinnamyl acetate	−99.4	−88.0	−108.9	−76.9	−80.6	−89.5
(*R*)-Citronellal	−99.9	−91.6	−105.0	−75.1	−73.3	−88.0
(*S*)-Citronellal	−98.3	−88.9	−107.4	−72.1	−71.4	−86.2
(*R*)-Citronellol	−99.9	−90.2	−104.9	−75.7	−72.3	−84.9
(*S*)-Citronellol	−99.2	−92.2	−107.6	−74.2	−77.4	−85.1
(*R*)-Citronellyl formate	−105.5	−92.6	−109.9	−72.8	−76.2	−90.5
(*S*)-Citronellyl formate	−101.5	−96.4	−114.7	−81.9	−78.3	−91.0
α-Copaene	−84.1	−78.9	−85.9	−60.2	−59.6	−77.1
Costunolide	−94.5	−99.2	−110.2	−78.2	−74.9	−92.6
(*R*)-Cryptone	−86.6	−82.4	−93.5	−68.2	−70.4	−74.8
(*S*)-Cryptone	−81.5	−82.9	−94.9	−69.8	−70.6	−76.6
β-Cubebene	−90.8	−92.0	−86.7	−76.0	−71.2	−89.8
Cuminaldehyde	−86.2	−80.5	−103.3	−74.9	−67.5	−81.7
*ar*-Curcumene	−105.3	−94.0	−108.6	−82.8	−75.8	−96.4
Curcumol	−83.0	−93.4	−91.2	−73.5	−69.6	−86.0
Curdione	−86.2	−98.1	−99.0	−77.3	−71.7	−92.0
Curzerene	−91.5	−86.0	−104.2	−76.8	−67.6	−90.5
*m*-Cymene	−83.6	−81.3	−92.8	−72.0	−67.6	−79.0
*o*-Cymene	−83.2	−78.3	−87.7	−63.9	−63.3	−72.9
*p*-Cymene	−79.7	−78.7	−91.0	−70.3	−63.9	−75.4
(*E*)-β-Damascenone	−85.2	−87.6	−110.2	−73.8	−73.8	−84.8
(2*E*,4*E*)-Decadienal	−105.7	−97.7	−112.5	−81.8	−78.7	−90.0
Dodecanal	−102.8	−93.9	−101.9	−77.9	−73.3	−94.9
Eremanthin	−97.7	−97.7	−98.0	−83.7	−81.3	−100.9
β-Eudesmol	−97.6	−87.4	−106.5	−75.8	−64.5	−74.7
γ-Eudesmol	−93.6	−89.9	−94.7	−69.4	−66.1	−84.3
Eugenol	−93.2	−91.7	−105.2	−80.0	−79.1	−88.4
Eugenol methyl ether	−88.5	−85.3	−111.1	−71.7	−68.6	−89.3
Eugenyl acetate	−96.3	−89.3	−115.1	−75.6	−70.6	−94.9
(*E*,*E*)-α-Farnesene	−115.0	−107.5	−112.8	−86.8	−85.3	−100.3
(*E*)-β-Farnesene	−115.4	−105.0	−116.3	−87.1	−82.9	−100.7
(*E*,*E*)-Farnesol	−112.4	−104.6	−121.4	−89.6	−80.8	−100.9
(+)-Fenchone	−80.0	−80.9	−87.1	−66.1	−60.8	−66.9
(-)-Fenchone	−83.3	−67.5	−86.2	−66.5	−60.9	−68.8
Flavesone	−82.3	−84.0	−95.7	−68.9	−64.4	−78.9
Geranial	−101.7	−90.8	−113.0	−76.1	−74.5	−92.0
Geraniol	−103.5	−98.5	−110.2	−77.4	−76.4	−93.8
Geranyl formate	−105.9	−93.9	−111.4	−80.8	−80.3	−96.2
Germacrene D	−92.1	−96.7	−110.5	−77.1	−73.0	−87.3
Germacrone	−85.1	−97.1	−94.9	−71.6	−67.1	−80.9
Guiaol	−94.1	−92.5	−113.4	−79.2	−79.4	−91.9
α-Gurjunene	−89.9	−83.2	−81.3	−71.5	−61.3	−79.8
β-Gurjunene	−77.4	−64.1	−81.1	−64.1	−58.0	−75.1
α-Himachalene	−80.4	−89.7	−86.5	−63.7	−67.9	−74.3
β-Himachalene	−85.5	−81.3	−88.6	−68.1	−62.9	−77.2
Himachalol	−77.2	−88.3	−100.8	−63.6	−63.7	−75.6
α-Humulene	−88.9	−90.9	−89.1	−74.9	−70.6	−86.7
Leptospermone	−85.5	−83.2	−92.9	−71.2	−64.1	−80.0
*iso*-Leptospermone	−86.1	−82.7	−93.7	−69.1	−62.6	−83.5
(*R*)-Limonene	−82.2	−76.2	−92.0	−72.6	−67.3	−79.0
(*S*)-Limonene	−82.2	−73.8	−92.2	−72.4	−66.1	−77.5
(*R*,*S*,*R*)-Limonene oxide	−86.3	−77.7	−100.8	−76.7	−68.1	−85.1
(*S*,*R*,*R*)-Limonene oxide	−83.5	−87.4	−89.4	−66.7	−63.4	−76.4
(*S*,*R*,*S*)-Limonene oxide	−84.7	−80.7	−95.8	−74.9	−66.7	−83.8
(*R*)-Linalool	−96.0	−89.3	−101.4	−70.9	−79.0	−87.0
(*S*)-Linalool	−100.7	−87.5	−102.1	−71.4	−70.4	−87.8
(*R*)-Linalyl acetate	−101.4	−88.9	−105.1	−73.0	−74.3	−82.5
(*S*)-Linalyl acetate	−102.8	−90.8	−106.0	−68.5	−74.5	−82.3
*p*-Mentha-1(7),8-diene	−82.3	−82.2	−94.3	−69.5	−65.9	−79.3
(+)-Menthol	−82.9	−86.2	−95.0	−71.8	−66.6	−77.7
(-)-Menthol	−82.3	−84.5	−95.1	−71.1	−69.2	−79.4
Menthone	−83.2	−77.8	−94.7	−69.3	−70.0	−74.4
*iso*-Menthone	−81.0	−79.8	−97.6	−64.4	−63.5	−80.1
α-Muurolene	−86.9	−81.9	−96.9	−72.0	−66.0	−83.7
(+)-γ-Muurolene	−82.3	−82.9	−83.1	−72.2	−66.6	−84.4
(-)-γ-Muurolene	−88.4	−86.2	−106.7	−74.5	−68.5	−86.8
Myrcene	−98.7	−90.1	−102.2	−74.9	−71.4	−84.3
Neral	−102.6	−91.8	−110.5	−81.5	−72.4	−91.2
(*E*)-Nerolidol	−110.7	−101.4	−113.8	−83.5	−76.1	−100.6
(*E*)-β-Ocimene	−97.0	−90.2	−103.6	−76.3	−75.6	−86.7
(*Z*)-β-Ocimene	−98.3	−88.7	−103.2	−78.6	−73.9	−85.1
Octanal	−89.7	−83.8	−99.5	−71.3	−72.4	−80.5
(*R*)-2-Octen-3-ol	−88.1	−88.4	−96.6	−71.9	−70.7	−81.9
(*S*)-1-Octen-3-ol	−94.2	−90.7	−93.4	−77.1	−74.7	−80.0
3-Octanone	−88.9	−85.3	−94.7	−71.7	−72.9	−80.3
Patchouli alcohol	−63.5	−57.1	−66.7	−51.8	−43.0	−67.9
(*R*)-Phellandral	−87.6	−85.9	−102.5	−74.4	−66.2	−81.1
(*S*)-Phellandral	−87.7	−83.7	−101.6	−74.4	−66.5	−79.2
(*R*)-α-Phellandrene	−81.1	−82.9	−92.5	−70.1	−65.3	−76.4
(*S*)-α-Phellandrene	−81.6	−82.5	−92.3	−69.7	−65.6	−76.6
(*R*)-β-Phellandrene	−84.4	−83.7	−94.0	−69.8	−65.5	−75.2
(*S*)-β-Phellandrene	−82.9	−84.5	−94.0	−71.2	−67.5	−77.7
Phytol	−106.3	−94.2	−118.9	−74.9	−75.6	−93.3
Phytone	−106.4	−94.3	−116.9	−79.8	−72.3	−90.4
(+)-α-Pinene	−79.0	−70.7	−79.7	−59.4	−54.6	−63.7
(-)-α-Pinene	−77.3	−70.4	−72.9	−61.3	−54.7	−63.6
(+)-β-Pinene	−76.8	−71.8	−79.4	−63.7	−53.0	−65.2
(-)-β-Pinene	−78.8	−73.6	−73.9	−61.9	−56.1	−64.7
(+)-Pinocamphone	−74.9	−72.6	−79.4	−63.2	−62.4	−66.4
(-)-Pinocamphone	−73.2	−79.3	−81.8	−62.0	−61.4	−67.2
(+)-*iso*-Pinocamphone	−74.9	−75.0	−74.9	−66.6	−55.1	−66.5
(-)-*iso*-Pinocamphone	−77.1	−80.7	−77.5	−64.0	−55.6	−68.3
(+)-*cis*-Pinocarveol	−73.9	−78.1	−79.5	−67.7	−57.4	−70.2
(-)-*cis*-Pinocarveol	−74.1	−76.5	−78.8	−67.0	−58.6	−66.3
(+)-*trans*-Pinocarveol	−74.7	−80.5	−78.7	−65.8	−57.2	−70.6
(-)-*trans*-Pinocarveol	−77.6	−80.0	−82.6	−60.5	−55.5	−70.8
(+)-Piperitone oxide	−82.0	−81.5	−98.5	−66.2	−65.2	−77.8
(-)-Piperitone oxide	−81.6	−83.9	−94.9	−68.6	−60.8	−82.8
(-)-*iso*-Pulegol	−82.5	−85.1	−98.8	−75.2	−66.7	−79.3
(*R*)-Pulegone	−84.0	−83.1	−96.1	−66.4	−65.5	−78.3
(*S*)-Pulegone	−83.6	−78.6	−93.3	−67.2	−66.2	−78.9
Rotundifolone	−83.1	−79.0	−96.6	−66.4	−62.1	−80.7
(+)-Sabinene	−86.4	−84.0	−92.2	−70.5	−68.6	−77.4
(-)-Sabinene	−87.8	−85.9	−94.7	−71.5	−69.6	−77.0
*cis*-Sabinene hydrate	−82.4	−81.1	−92.4	−68.0	−60.8	−78.2
(*Z*)-α-Santalol	−105.7	−95.3	−108.8	−84.7	−70.7	−95.8
(*E*)-β-Santalol	−104.8	−95.6	−106.2	−77.0	−70.9	−86.6
(*Z*)-β-Santalol	−104.4	−94.4	−106.2	−79.8	−73.2	−92.2
β-Sesquiphellandrene	−103.8	−99.0	−115.7	−84.8	−75.3	−101.1
Spathulenol	−90.7	−88.0	−98.4	−77.8	−67.9	−90.4
(*Z*)-Spiroether	−111.8	−84.9	−103.1	−87.1	−79.3	−102.0
γ-Terpinene	−81.3	−79.3	−93.2	−71.2	−65.3	−76.6
(*R*)-Terpinen-4-ol	−80.4	−81.9	−89.5	−69.0	−64.9	−75.3
(*S*)-Terpinen-4-ol	−82.1	−81.8	−88.0	−70.4	−64.4	−76.4
(*R*)-α-Terpineol	−82.6	−88.3	−91.6	−65.6	−66.0	−72.6
(*S*)-α-Terpineol	−88.7	−86.8	−94.1	−71.6	−63.5	−82.0
Terpinolene	−80.7	−80.1	−94.7	−68.8	−63.9	−77.4
(-)-α-Thujone	−87.2	−89.3	−94.5	−66.9	−69.2	−78.1
(+)-β-Thujone	−86.9	−80.5	−94.2	−73.7	−69.7	−79.2
Thymohydroquinone dimethyl ether	−89.5	−82.8	−104.6	−73.1	−67.0	−85.0
Thymol	−84.4	−87.5	−94.6	−72.9	−70.8	−78.4
2-Undecanone	−101.3	−94.0	−108.3	−78.4	−72.8	−90.2
Valencene	−84.7	−81.7	−92.6	−74.4	−68.3	−81.7
(+)-Verbenone	−84.7	−73.2	−82.5	−68.4	−54.8	−71.2
(-)-Verbenone	−83.8	−77.7	−77.4	−65.4	−57.8	−68.0
Viridiflorene	−86.8	−90.2	−91.0	−76.3	−69.4	−88.8
α-Zingiberene	−106.4	−100.5	−115.4	−82.7	−71.4	−98.6

^a^ Human angiotensin-converting enzyme 2 (hACE2).

**Table 4:** Normalized docking scores ( DS_norm_, kJ/mol) of essential oil components with bovine odorant binding protein (BtOBP), cruzain, torpedo acetylcholinesterase (TcAChE), *Bacillus anthracis* nicotinate mononucleotide adenylytransferase (BaNadD), Russell’s viper phospholipase A_2_ (DrPLA2), and *Escherichia coli* l-aspartate aminotransferase (EcAspTA).

Compound	BtOBP	Cruzain	TcAChE	BaNadD	DrPLA2	EcAspTA
	1GT3	1ME3	6G1U	3HFJ	1FV0	2Q7W
(*E*)-Anethole	−90.2	−73.3	−96.8	−112.9	−85.3	−88.1
*allo*-Aromadendrene	−85.0	−70.6	−92.8	−105.8	−87.8	−79.6
Artemiseole	−84.8	−74.2	−84.2	−90.6	−68.0	−71.7
(*R*)-Artemisia alcohol	−88.4	−68.3	−89.5	−94.8	−80.7	−81.5
(*S*)-Artemisia alcohol	−86.1	−74.0	−94.8	−101.4	−82.5	−81.4
Artemisia ketone	−93.1	−77.3	−91.5	−107.9	−87.8	−80.6
Ascaridole	−79.7	−54.4	−72.1	−79.7	−75.0	−65.3
Benzyl benzoate	−105.0	−82.8	−115.8	−128.1	−97.3	−89.9
(*Z*)-*trans*-α-Bergamotol	−1 00.7	−73.6	−107.0	−94.2	−92.6	−85.8
Bicyclogermacrene	−97.5	−82.4	−95.6	−105.5	−89.7	−89.0
Bicyclosesquiphellandrene	−92.2	−52.5	−91.2	−110.7	−88.4	−75.7
α-Bisabolol oxide A	−94.7	−66.1	−102.8	−102.6	−77.1	−82.7
α-Bisabolol oxide B	−104.6	−90.4	−107.0	−121.9	−94.2	−89.8
Bisabolone oxide A	−100.1	−71.9	−96.8	−84.7	−81.2	−86.7
(+)-Borneol	−72.5	−51.0	−72.0	−47.8	−61.4	−62.5
(-)-Borneol	−78.3	−53.1	−70.8	−49.0	−63.3	−56.2
(+)-*iso*-Borneol	−73.7	−55.6	−74.6	−31.0	−60.9	−59.7
(-)-*iso*-Borneol	−73.2	−55.3	−77.7	−52.6	−51.0	−57.4
α-Bulnesene	−93.4	−89.3	−109.3	−115.7	−94.4	−95.3
δ-Cadinene	−79.5	−86.8	−105.1	−116.8	−94.8	−82.3
Τ-Cadinol	−87.8	−71.8	−117.7	−121.7	−86.0	−87.0
(*R*)-Calamene	−80.3	−84.4	−116.5	−120.4	−91.3	−87.7
(*S*)-Calamene	−80.1	−82.5	−116.2	−121.8	−93.0	−88.3
(+)-Camphene	−79.0	−62.1	−77.0	−75.9	−69.3	−67.5
(-)-Camphene	−77.7	−62.8	−75.2	−77.4	−71.3	−70.6
(+)-Camphor	−76.0	−57.0	−75.0	−49.0	−53.5	−54.1
(-)-Camphor	−80.0	−54.1	−74.4	−52.9	−61.2	−56.4
(+)-δ-3-Carene	−80.4	−64.2	−80.6	−88.0	−71.8	−72.5
(-)-δ-3-Carene	−82.0	−68.9	−84.4	−90.2	−72.8	−72.9
Carvacrol	−82.4	−77.7	−94.8	−106.7	−81.6	−87.5
Carvacrol methyl ether	−85.6	−79.3	−96.6	−111.5	−84.2	−93.6
(+)-*cis*-Carveol	−89.1	−77.3	−96.9	−109.4	−81.3	−87.6
(-)-*cis*-Carveol	−89.0	−82.1	−95.5	−109.0	−83.2	−89.4
(*R*)-Carvone	−86.0	−80.7	−94.6	−109.9	−83.5	−91.5
(*S*)-Carvone	−88.0	−77.2	−94.1	−105.9	−82.8	−87.2
(*E*)-Caryophyllene	−87.3	−60.4	−88.1	−87.3	−87.4	−78.6
Caryophyllene oxide	−95.1	−73.1	−90.1	−97.0	−83.8	−77.8
Cedrol	−87.7	−63.1	−96.4	−84.4	−86.3	−70.9
*epi*-Cedrol	−84.0	−59.7	−83.3	−86.7	−80.0	−76.2
Chamazulene	−95.2	−89.2	−123.0	−132.1	−98.9	−101.6
(-)-*cis*-Chrystanthyl acetate	−85.8	−63.7	−89.8	−73.8	−76.5	−71.5
(-)-*trans*-Chrysanthenyl acetate	−80.9	−69.7	−82.4	−78.5	−76.5	−68.6
1,8-Cineole	−69.3	−51.1	−73.2	−54.4	−49.4	−47.3
(*E*)-Cinnamaldehyde	−87.7	−77.6	−98.2	−110.1	−85.7	−89.5
(*E*)-Cinnamyl acetate	−95.9	−88.2	−107.4	−132.5	−94.6	−103.1
(*R*)-Citronellal	−98.1	−90.5	−109.3	−120.7	−88.3	−105.7
(*S*)-Citronellal	−95.0	−89.7	−108.5	−123.3	−86.9	−104.1
(*R*)-Citronellol	−91.0	−90.0	−108.2	−122.4	−88.6	−107.7
(*S*)-Citronellol	−92.5	−91.9	−108.5	−122.0	−90.3	−100.3
(*R*)-Citronellyl formate	−98.0	−93.5	−118.7	−129.5	−87.5	−108.5
(*S*)-Citronellyl formate	−99.7	−95.1	−111.2	−135.6	−91.5	−94.8
α-Copaene	−88.9	−69.9	−84.6	−78.7	−81.7	−61.9
Costunolide	−106.8	−87.5	−116.7	−120.1	−100.3	−89.1
(*R*)-Cryptone	−74.9	−68.1	−92.2	−100.0	−78.5	−80.8
(*S*)-Cryptone	−79.0	−68.5	−91.6	−100.7	−82.7	−79.6
β-Cubebene	−99.8	−67.2	−106.9	−112.2	−93.9	−94.8
Cuminaldehyde	−84.9	−79.1	−95.3	−109.1	−90.1	−89.5
*ar*-Curcumene	−98.1	−87.5	−116.0	−125.7	−94.1	−95.3
Curcumol	−90.1	−67.6	−100.4	−88.0	−87.6	−81.5
Curdione	−96.4	−75.1	−99.3	−116.3	−91.0	−78.7
Curzerene	−96.4	−80.3	−109.4	−89.7	−91.1	−86.4
*m*-Cymene	−78.9	−73.1	−96.1	−99.1	−76.6	−88.0
*o*-Cymene	−77.0	−61.9	−89.8	−97.8	−76.2	−77.2
*p*-Cymene	−82.8	−73.2	−91.3	−96.8	−80.9	−84.3
(*E*)-β-Damascenone	−90.1	−80.5	−100.7	−105.4	−88.9	−80.0
(2*E*,4*E*)-Decadienal	−96.4	−87.0	−110.4	−129.7	−95.8	−114.8
Dodecanal	−95.8	−83.7	−110.3	−127.3	−89.2	−108.9
Eremanthin	−80.1	−87.9	−121.3	−132.4	−98.7	−91.8
β-Eudesmol	−95.2	−76.1	−98.6	−114.1	−85.3	−87.1
γ-Eudesmol	−94.9	−78.2	−106.1	−105.2	−89.7	−89.1
Eugenol	−90.4	−82.4	−103.9	−119.3	−87.3	−98.8
Eugenol methyl ether	−93.6	−78.1	−106.3	−116.8	−89.3	−94.4
Eugenyl acetate	−94.9	−83.4	−109.2	−126.1	−100.1	−104.6
(*E*,*E*)-α-Farnesene	−115.5	−103.7	−129.8	−131.8	−101.2	−111.4
(*E*)-β-Farnesene	−112.1	−103.2	−122.7	−131.8	−105.3	−108.6
(*E*,*E*)-Farnesol	−116.8	−96.2	−133.0	−135.6	−100.5	−109.0
(+)-Fenchone	−78.4	−60.5	−79.5	−79.8	−62.4	−69.9
(-)-Fenchone	−80.3	−61.5	−81.0	−84.2	−65.5	−60.7
Flavesone	−87.1	−56.0	−88.6	−92.1	−85.2	−80.3
Geranial	−96.5	−94.9	−111.7	−119.3	−92.1	−101.5
Geraniol	−95.5	−93.6	−109.7	−118.0	−94.3	−107.3
Geranyl formate	−100.2	−89.7	−115.4	−128.3	−94.8	−114.1
Germacrene D	−102.4	−88.8	−109.9	−116.5	−93.7	−90.0
Germacrone	−92.4	−68.3	−94.5	−102.9	−88.3	−80.0
Guiaol	−100.8	−88.5	−113.3	−107.5	−92.6	−94.0
α-Gurjunene	−80.6	−61.6	−84.6	−100.0	−83.6	−78.1
β-Gurjunene	−89.3	−38.8	−80.3	11.3	−77.6	−71.1
α-Himachalene	−89.2	−67.9	−83.9	−96.3	−87.6	−73.4
β-Himachalene	−81.9	−65.4	−96.1	−110.8	−91.4	−76.8
Himachalol	−91.5	−64.1	−81.2	−24.7	−81.7	−66.3
α-Humulene	−94.0	−80.0	−89.9	−113.6	−89.2	−82.5
Leptospermone	−89.6	−64.0	−92.2	−102.7	−88.8	−80.7
*iso*-Leptospermone	−92.8	−63.0	−90.1	−99.4	−86.2	−78.2
(*R*)-Limonene	−86.2	−75.7	−92.4	−99.6	−81.7	−86.8
(*S*)-Limonene	−84.9	−77.6	−93.4	−99.8	−83.3	−87.3
(*R*,*S*,*R*)-Limonene oxide	−87.1	−76.0	−93.2	−110.7	−84.9	−86.3
(*S*,*R*,*R*)-Limonene oxide	−84.6	−68.8	−85.0	−84.3	−76.2	−69.8
(*S*,*R*,*S*)-Limonene oxide	−91.2	−73.8	−97.4	−93.6	−85.1	−87.9
(*R*)-Linalool	−95.5	−89.9	−116.6	−112.4	−90.3	−89.9
(*S*)-Linalool	−93.0	−88.9	−110.1	−111.9	−91.0	−95.7
(*R*)-Linalyl acetate	−96.9	−87.1	−107.1	−112.5	−87.1	−95.2
(*S*)-Linalyl acetate	−100.0	−90.5	−110.2	−114.3	−86.6	−87.1
*p*-Mentha-1(7),8-diene	−83.3	−74.4	−93.6	−100.0	−82.9	−84.9
(+)-Menthol	−86.1	−66.4	−98.6	−106.1	−79.2	−90.8
(-)-Menthol	−81.0	−68.9	−97.5	−106.3	−80.5	−89.7
Menthone	−82.4	−72.0	−92.6	−98.2	−82.8	−83.5
*iso*-Menthone	−79.9	−64.8	−88.2	−102.4	−75.1	−82.0
α-Muurolene	−91.2	−79.9	−95.6	−113.7	−90.0	−82.8
(+)-γ-Muurolene	−85.2	−77.0	−98.7	−113.9	−93.2	−78.7
(-)-γ-Muurolene	−83.1	−83.4	−96.9	−106.3	−82.9	−80.5
Myrcene	−90.4	−88.8	−109.0	−112.7	−90.3	−101.1
Neral	−95.1	−94.1	−111.1	−122.8	−90.9	−105.4
(*E*)-Nerolidol	−111.5	−101.8	−131.9	−131.3	−99.6	−110.5
(*E*)-β-Ocimene	−91.8	−89.4	−104.6	−116.4	−89.0	−97.0
(*Z*)-β-Ocimene	−89.7	−90.6	−106.8	−112.1	−90.3	−94.4
Octanal	−85.8	−79.8	−97.6	−112.0	−85.7	−102.7
(*R*)-2-Octen-3-ol	−84.3	−81.5	−106.9	−107.1	−83.3	−101.1
(*S*)-1-Octen-3-ol	−84.5	−82.4	−108.4	−105.9	−85.0	−99.5
3-Octanone	−84.2	−82.5	−102.6	−111.6	−82.9	−101.8
Patchouli alcohol	−75.1	−41.4	−71.1	100.1	−10.1	−49.2
(*R*)-Phellandral	−92.3	−79.3	−97.4	−108.1	−88.9	−89.5
(*S*)-Phellandral	−92.9	−79.9	−96.1	−109.2	−88.9	−88.8
(*R*)-α-Phellandrene	−84.0	−69.7	−94.7	−100.8	−82.4	−83.8
(*S*)-α-Phellandrene	−84.3	−69.6	−93.5	−99.4	−82.6	−80.6
(*R*)-β-Phellandrene	−82.2	−69.9	−93.5	−102.0	−80.6	−84.1
(*S*)-β-Phellandrene	−83.8	−69.0	−93.2	−100.5	−80.2	−81.2
Phytol	−112.8	−93.8	−132.4	−134.7	−102.4	−112.9
Phytone	−117.8	−92.1	−124.5	−131.1	−99.7	−107.2
(+)-α-Pinene	−74.2	−58.0	−73.4	−62.4	−62.5	−54.4
(-)-α-Pinene	−72.8	−58.3	−70.4	−64.6	−61.7	−57.2
(+)-β-Pinene	−70.9	−57.8	−74.0	−64.1	−60.9	−55.5
(-)-β-Pinene	−73.5	−58.4	−73.1	−65.0	−63.7	−57.0
(+)-Pinocamphone	−76.5	−58.2	−73.6	−53.1	−55.4	−63.6
(-)-Pinocamphone	−76.6	−66.1	−76.5	−65.9	−72.3	−62.7
(+)-*iso*-Pinocamphone	−75.3	−59.0	−76.6	−54.0	−59.1	−58.1
(-)-*iso*-Pinocamphone	−76.2	−59.6	−76.7	−56.4	−65.8	−56.2
(+)-*cis*-Pinocarveol	−75.7	−58.2	−75.2	−51.7	−57.5	−58.1
(-)-*cis*-Pinocarveol	−75.7	−63.9	−73.5	−52.5	−66.3	−54.5
(+)-*trans*-Pinocarveol	−77.5	−60.6	−76.5	−71.1	−63.9	−61.4
(-)-*trans*-Pinocarveol	−72.4	−58.6	−77.2	−71.4	−56.3	−62.6
(+)-Piperitone oxide	−88.5	−70.5	−100.5	−104.2	−80.4	−78.0
(-)-Piperitone oxide	−86.0	−73.6	−99.6	−104.5	−80.7	−83.8
(-)-*iso*-Pulegol	−85.1	−72.0	−93.5	−106.7	−83.5	−93.8
(*R*)-Pulegone	−81.8	−69.9	−90.3	−103.9	−78.4	−74.4
(*S*)-Pulegone	−79.7	−69.6	−89.3	−99.9	−81.5	−76.4
Rotundifolone	−86.1	−72.1	−96.1	−100.0	−82.4	−72.1
(+)-Sabinene	−87.9	−66.6	−84.5	−92.1	−80.5	−78.2
(-)-Sabinene	−79.7	−68.4	−91.4	−96.0	−80.8	−75.8
*cis*-Sabinene hydrate	−88.1	−70.2	−84.5	−91.1	−72.3	−68.1
(*Z*)-α-Santalol	−108.5	−85.6	−113.4	−106.8	−96.8	−90.7
(*E*)-β-Santalol	−105.0	−90.2	−110.6	−113.3	−94.3	−94.0
(*Z*)-β-Santalol	−108.0	−85.3	−110.4	−110.9	−96.5	−92.6
β-Sesquiphellandrene	−103.9	−92.2	−116.6	−127.5	−99.4	−98.3
Spathulenol	−95.9	−94.8	−97.7	−102.8	−84.3	−102.2
(*Z*)-Spiroether	−112.8	−85.5	−124.3	−136.1	−101.5	−102.9
γ-Terpinene	−84.3	−74.9	−93.4	−98.7	−82.8	−85.9
(*R*)-Terpinen-4-ol	−87.3	−66.7	−81.5	−94.0	−80.2	−72.2
(*S*)-Terpinen-4-ol	−87.1	−67.2	−78.9	−92.3	−80.2	−71.7
(*R*)-α-Terpineol	−82.0	−68.0	−80.3	−82.2	−77.6	−71.3
(*S*)-α-Terpineol	−76.9	−75.5	−103.6	−101.7	−86.6	−84.9
Terpinolene	−83.1	−69.8	−89.0	−101.8	−84.8	−72.9
(-)-α-Thujone	−81.4	−66.2	−84.9	−93.4	−72.6	−74.1
(+)-β-Thujone	−87.6	−66.4	−89.9	−102.9	−71.7	−86.9
Thymohydroquinone dimethyl ether	−86.3	−75.8	−103.7	−116.0	−85.3	−95.5
Thymol	−84.4	−70.8	−96.4	−107.1	−79.7	−91.8
2-Undecanone	−94.5	−90.2	−110.0	−129.7	−91.9	−107.6
Valencene	−96.7	−75.1	−103.3	−114.7	−87.8	−76.6
(+)-Verbenone	−74.3	−64.5	−79.3	−71.6	−63.9	−72.3
(-)-Verbenone	−73.5	−63.1	−78.0	−65.3	−64.8	−59.3
Viridiflorene	−81.9	−68.6	−102.1	−106.9	−73.1	−81.9
α-Zingiberene	−108.3	−90.9	−113.8	−123.4	−97.7	−99.9

## Abbreviations

Adeno	Adenovirus
AIV	Avian influenza virus
BaNadD	*Bacillus anthracis* nicotinate mononucleotide adenylytransferase
BtOBP	Bovine odorant binding protein
COVID-19	Coronavirus disease 2019
Cox B1	Coxsackie B1 virus
DENV-1	Dengue virus serotype 1
DENV-2	Dengue virus serotype 2
DENV-3	Dengue virus serotype 3
DENV-4	Dengue virus serotype 4
DrPLA2	Russell’s viper phospholipase A_2_
DS_norm_	Normalized docking score
EcAspTA	Esherichia coli L-aspartate aminotransferase
ECHO 9	Echovirus 9
hACE2	Human angiotensin-converting enzyme
HeLa	Human cervical tumor cells
HEp-2	Human epithelial type 2 cells
HRSV	Human respiratory syncytial virus
HSV-1	Herpes simplex virus 1
HSV-2	Herpes simplex virus 2
IC_50_	Median inhibitory concentration
JUNV	Junin virus
MDCK	Madin-Darby canine kidney cells
MTT	3-(4,5-dimethylthiazol-2-yl)-2,5-diphenyltetrazolium bromide
PCR	Polymerase chain reaction
PDB	Protein data bank
Polio 1	Poliomyelitis virus 1
RC-37	African green monkey kidney cells
SARS-CoV-2	2019 severe acute respiratory syndrome coronavirus 2
TcAChE	Torpedo acetylcholinesterase
Vero	African green monkey kidney cells
YFV	Yellow fever virus

## References

[1] Lai C.-C., Shih T.-P., Ko W.-C., Tang H.-J., Hsueh P.-R. (2020). Severe acute respiratory syndrome coronavirus 2 (SARS-CoV-2) and coronavirus disease-2019 (COVID-19): The epidemic and the challenges. Int. J. Antimicrob. Agents.

[2] Yang X., Yu Y., Xu J., Shu H., Xia J., Liu H., Wu Y., Zhang L., Yu Z., Fang M. (2020). Clinical course and outcomes of critically ill patients with SARS-CoV-2 pneumonia in Wuhan, China: A single-centered, retrospective, observational study. Lancet Respir. Med..

[3] WHO Coronavirus disease 2019 (COVID-19) Situation Report—94. https://www.who.int/emergencies/diseases/novel-coronavirus-2019/situation-reports/.

[4] Glezen W.P., Couch R.B., Evans A.S., Kaslow R.A. (1997). Influenza viruses. Viral Infections of Humans.

[5] Baigent S.J., McCauley J.W. (2003). Influenza type A in humans, mammals and birds: Determinants of virus virulence, host-range and interspecies transmission. BioEssays.

[6] Guan Y., Vijaykrishna D., Bahl J., Zhu H., Wang J., Smith G.J.D. (2010). The emergence of pandemic influenza viruses. Protein Cell.

[7] Johnson N.P.A.S., Mueller J. (2002). Updating the accounts: Global mortality of the 1918-1920 “Spanish” influenza pandemic. Bull. Hist. Med..

[8] Schnitzler S.U., Schnitzler P. (2009). An update on swine-origin influenza virus A/H1N1: A review. Virus Genes.

[9] Hsieh Y.-C., Wu T.-Z., Liu D.-P., Shao P.-L., Chang L.-Y., Lu C.Y., Lee C.Y., Huang F.-Y., Huang L.-M. (2006). Influenza pandemics: Past, present and future. J. Formos. Med. Assoc..

[10] Gauthier-Clerc M., Lebarbenchon C., Thomas F. (2007). Recent expansion of highly pathogenic avian influenza H5N1: A critical review. Ibis.

[11] Earn D.J.D., Dushoff J., Levin S.A. (2002). Ecology and evolution of the flu. Trends Ecol. Evol..

[12] Vimalanathan S., Hudson J. (2014). Anti-influenza virus activity of essential oils and vapors. Am. J. Essent. Oils Nat. Prod..

[13] Mallavarapu G.R., Ramesh S., Chandrasekhara R.S., Rao B.R.R., Kaul P.N., Bhattacharya A.K. (1995). Investigation of the essential oil of cinnamon leaf grown at Bangalore and Hyderabad. Flavour Fragr. J..

[14] Raina V.K., Srivastava S.K., Aggarwal K.K., Ramesh S., Kumar S. (2001). Essential oil composition of *Cinnamomum zeylanicum* Blume leaves from Little Andaman, India. Flavour Fragr. J..

[15] Fichi G., Flamini G., Zaralli L.J., Perrucci S. (2007). Efficacy of an essentifal oil of *Cinnamomum zeylanicum* against Psoroptes cuniculi. Phytomedicine.

[16] Verzera A., Trozzi A., Gazea F., Cicciarello G., Cotroneo A. (2003). Effects of rootstock on the composition of bergamot (*Citrus bergamia* Risso et Poiteau) essential oil. J. Agric. Food Chem..

[17] Sawamura M., Onishi Y., Ikemoto J., Tu N.T.M., Phi N.T.L. (2006). Characteristic odour components of bergamot (*Citrus bergamia* Risso) essential oil. Flavour Fragr. J..

[18] Costa R., Dugo P., Navarra M., Raymo V., Dugo G., Mondello L. (2008). Study on the chemical composition variability of some processed bergamot (*Citrus bergamia*) essential oils. Flavour Fragr. J..

[19] Schipilliti L., Dugo G., Santi L., Dugo P., Mondello L. (2011). Authentication of bergamot essential oil by gas chromatography-combustion-isotope ratio mass spectrometer (GC-C-IRMS). J. Essent. Oil Res..

[20] Tundis R., Loizzo M.R., Bonesi M., Menichini F., Mastellone V., Colica C., Menichini F. (2012). Comparative study on the antioxidant capacity and cholinesterase inhibitory activity of *Citrus aurantifolia* Swingle, *C. aurantium* L., and *C. bergamia* Risso and Poit. peel essential oils. J. Food Sci..

[21] Padalia R.C., Verma R.S., Chanotiya C.S., Yadav A. (2011). Chemical fingerprinting of the fragrant volatiles of nineteen Indian cultivars of *Cymbopogon* Spreng. (Poaceae). Rec. Nat. Prod..

[22] Gupta A.K., Muhury R., Ganjewala D. (2016). A study on antimicrobial activities of essential oils of different cultivars of lemongrass (*Cymbopogon flexuosus*). Pharm. Sci..

[23] Satyal P., Murray B.L., McFeeters R.L., Setzer W.N. (2016). Essential oil characterization of *Thymus vulgaris* from various geographical locations. Foods.

[24] Erdoǧan Orhan I., Özçelik B., Kartal M., Kan Y. (2012). Antimicrobial and antiviral effects of essential oils from selected Umbelliferae and Labiatae plants and individual essential oil components. Turkish J. Biol..

[25] Alburn H.E., Chester W., Greenspan G. (1972). Thymol as an anti-influenza agent. U.S. Patent.

[26] Evandri M.G., Battinelli L., Daniele C., Mastrangelo S., Bolle P., Mazzanti G. (2005). The antimutagenic activity of Lavandula angustifolia (lavender) essential oil in the bacterial reverse mutation assay. Food Chem. Toxicol..

[27] De Martino L., De Feo V., Nazzaro F. (2009). Chemical composition and in vitro antimicrobial and mutagenic activities of seven Lamiaceae essential oils. Molecules.

[28] Lafhal S., Vanloot P., Bombarda I., Kister J., Dupuy N. (2016). Chemometric analysis of French lavender and lavandin essential oils by near infrared spectroscopy. Ind. Crops Prod..

[29] De Rapper S., Viljoen A., van Vuuren S. (2016). The in vitro antimicrobial effects of *Lavandula angustifolia* essential oil in combination with conventional antimicrobial agents. Evidence-Based Complement. Altern. Med..

[30] Hammer K.A., Carson C.F., Riley T.V., Nielsen J.B. (2006). A review of the toxicity of *Melaleuca alternifolia* (tea tree) oil. Food Chem. Toxicol..

[31] Garozzo A., Timpanaro R., Bisignano B., Furneri P.M., Bisignano G., Castro A. (2009). In vitro antiviral activity of *Melaleuca alternifolia* essential oil. Lett. Appl. Microbiol..

[32] Garozzo A., Timpanaro R., Stivala A., Bisignano G., Castro A. (2011). Activity of *Melaleuca alternifolia* (tea tree) oil on influenza virus A/PR/8: Study on the mechanism of action. Antiviral Res..

[33] Usachev E.V., Pyankov O.V., Usacheva O.V., Agranovski I.E. (2013). Antiviral activity of tea tree and eucalyptus oil aerosol and vapour. J. Aerosol Sci..

[34] Sonnberg S., Webby R.J., Webster R.G. (2013). Natural history of highly pathogenic avian influenza H5N1. Virus Res..

[35] Belser J.A., Tumpey T.M. (2013). H5N1 pathogenesis studies in mammalian models. Virus Res..

[36] Nagy M.M., Al-Mahdy D.A., Abd El Aziz O.M., Kandil A.M., Tantawy M.A., El Alfy T.S.M. (2018). Chemical composition and antiviral activity of essential oils from *Citrus reshni* Hort. ex Tanaka (Cleopatra mandarin) cultivated in Egypt. J. Essent. Oil-Bearing Plants.

[37] García C.C., Talarico L., Almeida N., Colombres S., Duschatzky C., Damonte E.B. (2003). Virucidal activity of essential oils from aromatic plants of San Luis, Argentina. Phyther. Res..

[38] Duschatzky C.B., Possetto M.L., Talarico L.B., García C.C., Michis F., Almeida N.V., De Lampasona M.P., Schuff C., Damonte E.B. (2005). Evaluation of chemical and antiviral properties of essential oils from South American plants. Antivir. Chem. Chemother..

[39] Sinico C., De Logu A., Lai F., Valenti D., Manconi M., Loy G., Bonsignore L., Fadda A.M. (2005). Liposomal incorporation of *Artemisia arborescens* L. essential oil and in vitro antiviral activity. Eur. J. Pharm. Biopharm..

[40] Saddi M., Sanna A., Cottiglia F., Chisu L., Casu L., Bonsignore L., De Logu A. (2007). Antiherpevirus activity of *Artemisia arborescens* essential oil and inhibition of lateral diffusion in Vero cells. Ann. Clin. Microbiol. Antimicrob..

[41] Gavanji S., Sayedipour S.S., Larki B., Bakhtari A. (2015). Antiviral activity of some plant oils against herpes simplex virus type 1 in Vero cell culture. J. Acute Med..

[42] Chung M.S. (2017). Antiviral activities of Artemisia princeps var. orientalis essential oil and its α-thujone against norovirus surrogates. Food Sci. Biotechnol..

[43] Meneses R., Ocazionez R.E., Martínez J.R., Stashenko E.E. (2009). Inhibitory effect of essential oils obtained from plants grown in Colombia on yellow fever virus replication in vitro. Ann. Clin. Microbiol. Antimicrob..

[44] Haddad J.G., Picard M., Bénard S., Desvignes C., Desprès P., Diotel N., El Kalamouni C. (2019). *Ayapana triplinervis* essential oil and its main component thymohydroquinone dimethyl ether inhibit Zika virus at doses devoid of toxicity in zebrafish. Molecules.

[45] Loizzo M.R., Saab A., Tundis R., Statti G.A., Lampronti I., Menichini F., Gambari R., Cinatl J., Doerr H.W. (2008). Phytochemical analysis and in vitro evaluation of the biological activity against herpes simplex virus type 1 (HSV-1) of *Cedrus libani* A. Rich. Phytomedicine.

[46] Setzer W.N. (2016). Essential oils as complementary and alternative medicines for the treatment of influenza. Am. J. Essent. Oil Nat. Prod..

[47] Bourne K.Z., Bourne N., Reising S.F., Stanberry L.R. (1999). Plant products as topical microbicide candidates: Assessment of in vitro and in vivo activity against herpes simplex virus type 2. Antiviral Res..

[48] Minami M., Kita M., Nakaya T., Yamamoto T., Kuriyama H., Imanishi J. (2003). The inhibitory effect of essential oils on herpes simplex virus type-1 replication in vitro. Microbiol. Immunol..

[49] El Mokni R., Youssef F.S., Jmii H., Khmiri A., Bouazzi S., Jlassi I., Jaidane H., Dhaouadi H., Ashour M.L., Hammami S. (2019). The Essential oil of Tunisian *Dysphania ambrosioides* and its antimicrobial and antiviral properties. J. Essent. Oil-Bearing Plants.

[50] Elaissi A., Rouis Z., Salem N.A.B., Mabrouk S., ben Salem Y., Salah K.B.H., Aouni M., Farhat F., Chemli R., Harzallah-Skhiri F. (2012). Chemical composition of 8 Eucalyptus species’ essential oils and the evaluation of their antibacterial, antifungal and antiviral activities. BMC Complement. Altern. Med..

[51] El-Baz F.K., Mahmoud K., El-Senousy W.M., Darwesh O.M., El Gohary A.E. (2015). Antiviral – antimicrobial and schistosomicidal activities of *Eucalyptus camaldulensis* essential oils. Int. J. Pharm. Sci. Rev. Res..

[52] Astani A., Reichling J., Schnitzler P. (2010). Comparative study on the antiviral activity of selected monoterpenes derived from essential oils. Phyther. Res..

[53] Ibrahim N.A., El-Hawary S.S., Mohammed M.M.D., Farid M.A., Abdel-Wahed N.A.M., Ali M.A., El-Abd E.A.W. (2015). Chemical composition, antiviral against avian influenza (H5N1) virus and antimicrobial activities of the essential oils of the leaves and fruits of *Fortunella margarita* Lour. Swingle growing in Egypt. J. Appl. Pharm. Sci..

[54] Hayashi K., Kamiya M., Hayashi T. (1995). Virucidal effects of the steam distillate from *Houttuynia cordata* and its components on HSV-1, influenza virus, and HIV. Planta Med..

[55] Brand Y.M., Roa-Linares V.C., Betancur-Galvis L.A., Durán-García D.C., Stashenko E. (2016). Antiviral activity of Colombian Labiatae and Verbenaceae family essential oils and monoterpenes on human herpes viruses. J. Essent. Oil Res..

[56] Schnitzler P., Koch C., Reichling J. (2007). Susceptibility of drug-resistant clinical herpes simplex virus type 1 strains to essential oils of ginger, thyme, hyssop, and sandalwood. Antimicrob. Agents Chemother..

[57] Koch C., Reichling J., Schneele J., Schnitzler P. (2008). Inhibitory effect of essential oils against herpes simplex virus type 2. Phytomedicine.

[58] Koch C., Reichling J., Kehm R., Sharaf M.M., Zentgraf H., Schneele J., Schnitzler P. (2008). Efficacy of anise oil, dwarf-pine oil and chamomile oil against thymidine-kinase-positive and thymidine-kinase-negative herpesviruses. J. Pharm. Pharmacol..

[59] Astani A., Schnitzler P., Reichling J. (2011). Screening for antiviral activities of isolated compounds from essential oils. Evidence-Based Complement. Altern. Med..

[60] Reichling J., Koch C., Stahl-Biskup E., Sojka C., Schnitzler P. (2005). Virucidal activity of a β-triketone-rich essential oil of *Leptospermum scoparium* (Manuka Oil) Against HSV-1 and HSV-2 in cell culture. Planta Med..

[61] Ocazionez R.E., Meneses R., Torres F.Á., Stashenko E. (2010). Virucidal activity of Colombian *Lippia* essential oils on dengue virus replication in vitro. Mem. Inst. Oswaldo Cruz.

[62] Gómez L.A., Stashenko E., Ocazionez R.E. (2013). Comparative study on in vitro activities of citral, limonene and essential oils from *Lippia citriodora* and *L. alba* on yellow fever virus. Nat. Prod. Commun..

[63] Pilau M.R., Alves S.H., Weiblen R., Arenhart S., Cueto A.P., Lovato L.T. (2011). Antiviral activity of the *Lippia graveolens* (Mexican oregano) essential oil and its main compound carvacrol against human and animal viruses. Brazilian J. Microbiol..

[64] Civitelli L., Panella S., Marcocci M.E., De Petris A., Garzoli S., Pepi F., Vavala E., Ragno R., Nencioni L., Palamara A.T. (2014). In vitro inhibition of herpes simplex virus type 1 replication by *Mentha suaveolens* essential oil and its main component piperitenone oxide. Phytomedicine.

[65] Brun P., Bernabè G., Filippini R., Piovan A. (2019). In vitro antimicrobial activities of commercially available tea tree (*Melaleuca alternifolia*) essential oils. Curr. Microbiol..

[66] Farag R.S., Shalaby A.S., El-Baroty G.A., Ibrahim N.A., Ali M.A., Hassan E.M. (2004). Chemical and biological evaluation of the essential oils of different *Melaleuca* species. Phyther. Res..

[67] Allahverdiyev A., Duran N., Ozguven M., Koltas S. (2004). Antiviral activity of the volatile oils of *Melissa officinalis* L. against Herpes simplex virus type-2. Phytomedicine.

[68] Schnitzler P., Schuhmacher A., Astani A., Reichling J. (2008). *Melissa officinalis* oil affects infectivity of enveloped herpesviruses. Phytomedicine.

[69] Schuhmacher A., Reichling J., Schnitzler P. (2003). Virucidal effect of peppermint oil on the enveloped viruses herpes simplex virus type 1 and type 2 in vitro. Phytomedicine.

[70] El Moussaoui N., Sanchez G., Khay E.O., Idaomar M., Ibn Mansour A., Abrini J., Aznar R. (2013). Antibacterial and antiviral activities of essential oils of northern Moroccan plants. Br. Biotechnol. J..

[71] Bouazzi S., Jmii H., El Mokni R., Faidi K., Falconieri D., Piras A., Jaïdane H., Porcedda S., Hammami S. (2018). Cytotoxic and antiviral activities of the essential oils from Tunisian fern, *Osmunda regalis*. S. Afr. J. Bot..

[72] Sivropoulou A., Nikolaou C., Papanikolaou E., Kokkini S., Lanaras T., Arsenakis M. (1997). Antimicrobial, cytotoxic, and antiviral activities of *Salvia fructicosa* essential oil. J. Agric. Food Chem..

[73] Benencia F., Courrèges M.C. (1999). Antiviral activity of sandalwood oil against herpes simplex viruses-1 and -2. Phytomedicine.

[74] De Logu A., Loy G., Pellerano M.L., Bonsignore L., Schivo M.L. (2000). Inactivation of HSV-1 and HSV-2 and prevention of cell-to-cell virus spread by *Santolina insularis* essential oil. Antiviral Res..

[75] Zeghib A., Calliste C.-A., Simon A., Charfeddine R., Aouni M., Duroux J.-L., Kabouche A., Kabouche Z. (2019). Chemical composition and biological potential of *Thymus willdenowii* Boiss. & Reut. essential oil. Nat. Prod. Res..

[76] Roy S., Chaurvedi P., Chowdhary A. (2015). Evaluation of antiviral activity of essential oil of *Trachyspermum ammi* against Japanese encephalitis virus. Pharmacognosy Res..

[77] Shayeganmehr A., Vasfi Marandi M., Karimi V., Barin A., Ghalyanchilangeroudi A. (2018). *Zataria multiflora* essential oil reduces replication rate of avian influenza virus (H9N2 subtype) in challenged broiler chicks. Br. Poult. Sci..

[78] Camero M., Lanave G., Catella C., Capozza P., Gentile A., Fracchiolla G., Britti D., Martella V., Buonavoglia C., Tempesta M. (2019). Virucidal activity of ginger essential oil against caprine alphaherpesvirus-1. Vet. Microbiol..

[79] Trovati G., Chierice G.O., Sanches E.A., Galhiane M.S. (2009). Essential oil composition of *Aloysia gratissima* from Brazil. J. Essent. Oil Res..

[80] Santos F.M., Pinto J.E.B.P., Bertolucci S.K.V., Alvarenga A.A., Alves M.N., Duarte M.C.T., Sartoratto A. (2013). Chemical composition and antimicrobial activity of the essential oil from the leaves and flowers of *Aloysia gratissima*. Rev. Bras. Plantas Med..

[81] Militello M., Settanni L., Aleo A., Mammina C., Moschetti G., Giammanco G.M., Blàzquez M.A., Carrubba A. (2011). Chemical composition and antibacterial potential of *Artemisia arborescens* L. essential oil. Curr. Microbiol..

[82] Bertuzzi G., Tirillini B., Angelini P., Venanzoni R. (2013). Antioxidative action of *Citrus limonum* essential oil on skin. European J. Med. Plants.

[83] El-Leel O.F.A., El-Said N.A.M. (2016). Growth, essential oil and molecular genetic identification studies of some *Eucalyptus* species cultivated under Egyptian conditions. BAOJ Biotechnol..

[84] Silvestre A.J.D., Cavaleiro J.A.S., Delmond B., Filliatre C., Bourgeois G. (1997). Analysis of the variation of the essential oil composition of *Eucalyptus globulus* Labill. from Portugal using multivariate statistical analysis. Ind. Crops Prod..

[85] Barazandeh M.M. (2002). Essential oil composition of *Lavandula latifolia* Medik from Iran. J. Essent. Oil Res..

[86] Pozzatti P., Scheid L.A., Spader T.B., Atayde M.L., Santurio J.M., Alves S.H. (2008). In vitro activity of essential oils extracted from plants used as spices against fluconazole-resistant and fluconazole-susceptible *Candida* spp.. Can. J. Microbiol..

[87] Raal A., Orav A., Püssa T., Valner C., Malmiste B., Arak E. (2012). Content of essential oil, terpenoids and polyphenols in commercial chamomile (*Chamomilla recutita* L. Rauschert) teas from different countries. Food Chem..

[88] Demir H., Kalaycı S. (2017). Chemical composition and antimicrobial activity of essential oils of *Ocimum basilicum* var. *album* (L.) Benth, *Lavandula angustifolia* subsp. *angustifolia*, *Melissa officinalis* belonging to Lamiaceae family. J. Food Sci. Eng..

[89] Stevanovic T., Garneau F.-X., Jean F.-I., Gagnon H., Vilotic D., Petrovic S., Ruzic N., Pichette A. (2005). The essential oil composition of *Pinus mugo* Turra from Serbia. Flavour Fragr. J..

[90] Hoim Y., Hiltunen R. (1999). Chemical composition of a commercial oil of *Ravensara aromatica* Sonn. used in aromatherapy. J. Essent. Oil Res..

[91] Poli F., Bonsignore L., Loy G., Sacchetti G., Ballero M. (1997). Comparison between the essential oils of *Santolina insularis* (Genn. ex Fiori) Arrigoni and *Santolina corsica* Jord. et Fourr. from the island of Sardinia (Italy). J. Ethnopharmacol..

[92] Tsai J.-J., Liu W.-L., Lin P.-C., Huang B.-Y., Tsai C.-Y., Chou P.-H., Lee F.-C., Ping C.-F., Lee P.-Y.A., Liu L.-T. (2019). An RT-PCR panel for rapid serotyping of dengue virus serotypes 1 to 4 in human serum and mosquito on a field-deployable PCR system. PLoS ONE.

[93] Vasconcelos P.F.C., Monath T.P. (2016). Yellow fever remains a potential threat to public health. Vector-Borne Zoonotic Dis..

[94] Douam F., Ploss A. (2018). Yellow fever virus: Knowledge gaps impeding the fight against an old foe. Trends Microbiol..

[95] Hayashi K., Imanishi N., Kashiwayama Y., Kawano A., Terasawa K., Shimada Y., Ochiai H. (2007). Inhibitory effect of cinnamaldehyde, derived from Cinnamomi cortex, on the growth of influenza A/PR/8 virus in vitro and in vivo. Antiviral Res..

[96] Benencia F., Courrèges M.C. (2000). In vitro and in vivo activity of eugenol on human herpesvirus. Phyther. Res..

[97] Liao Q., Qian Z., Liu R., An L., Chen X. (2013). Germacrone inhibits early stages of influenza virus infection. Antiviral Res..

[98] Armaka M., Papanikolaou E., Sivropoulou A., Arsenakis M. (1999). Antiviral properties of isoborneol, a potent inhibitor of herpes simplex virus type 1. Antiviral Res..

[99] Ryabchenko B., Tulupova E., Schmidt E., Wlcek K., Buchbauer G., Jirovetz L. (2008). Investigation of anticancer and antiviral properties of selected aroma samples. Nat. Prod. Commun..

[100] Kiyohara H., Ichino C., Kawamura Y., Nagai T., Sato N., Yamada H. (2012). Patchouli alcohol: In vitro direct anti-influenza virus sesquiterpene in *Pogostemon cablin* Benth. J. Nat. Med..

[101] Wu H., Li B., Wang X., Jin M., Wang G. (2011). Inhibitory effect and possible mechanism of action of patchouli alcohol against influenza a (H2N2) virus. Molecules.

[102] Gentile D., Patamia V., Scala A., Sciortino M.T., Piperno A., Rescifina A. (2020). Inhibitors of SARS-CoV-2 main protease from a library of marine natural products: A virtual screening and molecular modeling study. Mar. Drugs.

[103] Thuy B.T.P., My T.T.A., Hai N.T.T., Hieu L.T., Hoa T.T., Thi Phuong Loan H., Triet N.T., Van Anh T.T., Quy P.T., Van Tat P. (2020). Investigation into SARS-CoV-2 resistance of compounds in garlic essential oil. ACS Omega.

[104] Joshi R., Jagdale S., Bansode S., Shankar S.S., Tellis M., Pandya V.K., Giri A., Kulkarni M. (2020). Discovery of multi-target-directed ligands by targeting host-specific SARS-CoV-2’s structurally conserved main protease. Preprints.

[105] Manish M. (2020). Studies on computational molecular interaction between SARS-CoV-2 main protease and natural products. ChemRxiv.

[106] Beck B.R., Shin B., Choi Y., Park S., Kang K. (2020). Predicting commercially available antiviral drugs that may act on the novel coronavirus (SARS-CoV-2) through a drug-target interaction deep learning model. Comput. Struct. Biotechnol. J..

[107] Hofmarcher M., Mayr A., Rumetshofer E., Ruch P., Renz P., Schimunek J., Seidl P., Vall A., Widrich M., Hochreiter S. (2020). Large-scale ligand-based virtual screening for SARS-CoV-2 inhibitors using deep neural networks. SSRN Electron. J..

[108] Zhang L., Lin D., Sun X., Curth U., Drosten C., Sauerhering L., Becker S., Rox K., Hilgenfeld R. (2020). Crystal structure of SARS-CoV-2 main protease provides a basis for design of improved α-ketoamide inhibitors. Science.

[109] Bhardwaj K., Sun J., Holzenburg A., Guarino L.A., Kao C.C. (2006). RNA recognition and cleavage by the SARS coronavirus endoribonuclease. J. Mol. Biol..

[110] Saikatendu K.S., Joseph J.S., Subramanian V., Clayton T., Griffith M., Moy K., Velasquez J., Neuman B.W., Buchmeier M.J., Stevens R.C. (2005). Structural basis of severe acute respiratory syndrome coronavirus ADP-ribose-1″-phosphate dephosphorylation by a conserved domain of nsP3. Structure.

[111] Putics A., Filipowicz W., Hall J., Gorbalenya A.E., Ziebuhr J. (2005). ADP-ribose-1"-monophosphatase: A conserved coronavirus enzyme that is dispensable for viral replication in tissue culture. J. Virol..

[112] Chen S., Luo H., Chen L., Chen J., Shen J., Zhu W., Chen K., Shen X., Jiang H. (2006). An overall picture of SARS coronavirus (SARS-CoV) genome-encoded major proteins: Structures, functions and drug development. Curr. Pharm. Des..

[113] Yan R., Zhang Y., Li Y., Xia L., Guo Y., Zhou Q. (2020). Structural basis for the recognition of SARS-CoV-2 by full-length human ACE2. Science.

[114] Zhang H., Penninger J.M., Li Y., Zhong N., Slutsky A.S. (2020). Angiotensin-converting enzyme 2 (ACE2) as a SARS-CoV-2 receptor: Molecular mechanisms and potential therapeutic target. Intensive Care Med..

[115] Pourghanbari G., Nili H., Moattari A., Mohammadi A., Iraji A. (2016). Antiviral activity of the oseltamivir and *Melissa officinalis* L. essential oil against avian influenza A virus (H9N2). VirusDisease.

[116] Cowan M.M. (1999). Plant products as antimicrobial agents. Clin. Microbiol. Rev..

[117] Peana A.T., D’Aquila P.S., Chessa M.L., Moretti M.D.L., Serra G., Pippia P. (2003). (-)-Linalool produces antinociception in two experimental models of pain. Eur. J. Pharmacol..

[118] Li X.-J., Yang Y.-J., Li Y.-S., Zhang W.K., Tang H.-B. (2016). α-Pinene, linalool, and 1-octanol contribute to the topical anti-inflammatory and analgesic activities of frankincense by inhibiting COX-2. J. Ethnopharmacol..

[119] Bakir B., Him A., Özbek H., Düz E., Tütüncü M. (2008). Investigation of the anti-inflammatory and analgesic activities of β-caryophyllene. Int. J. Essent. Oil Ther..

[120] Klauke A., Racz I., Pradier B., Markert A., Zimmer A.M., Gertsch J., Zimmer A. (2014). The cannabinoid CB2 receptor-selective phytocannabinoid beta-caryophyllene exerts analgesic effects in mouse models of inflammatory and neuropathic pain. Eur. Neuropsychopharmacol..

[121] Santos F.A., Rao V.S.N. (2000). Antiinflammatory and antinociceptive effects of 1,8-cineole a terpenoid oxide present in many plant essential oils. Phyther. Res..

[122] Takaishi M., Fujita F., Uchida K., Yamamoto S., Shimizu M.S., Uotsu C.H., Shimizu M., Tominaga M. (2012). 1,8-Cineole, a TRPM8 agonist, is a novel natural antagonist of human TRPA1. Mol. Pain.

[123] Eccles R. (1994). Menthol and related cooling compounds. J. Pharm. Pharmacol..

[124] Kamatou G.P.P., Vermaak I., Viljoen A.M., Lawrence B.M. (2013). Menthol: A simple monoterpene with remarkable biological properties. Phytochemistry.

[125] Laude E.A., Morice A.H., Grattan T.J. (1994). The antitussive effects of menthol, camphor, and cineole in conscious Guinea-pigs. Pulm. Pharmacol..

[126] Gavliakova S., Dolak T., Licha H., Krizova S., Plevkova J. (2013). Cineole, thymol and camphor nasal challenges and their effect on nasal symptoms and cough in an animal model. Acta Medica Martiniana.

[127] Gavliakova S., Biringerova Z., Buday T., Brozmanova M., Calkovsky V., Poliacek I., Plevkova J. (2013). Antitussive effects of nasal thymol challenges in healthy volunteers. Respir. Physiol. Neurobiol..

[128] Snow Setzer M., Sharifi-Rad J., Setzer W.N. (2016). The search for herbal antibiotics: An in-silico investigation of antibacterial phytochemicals. Antibiotics.

[129] Setzer M.S., Byler K.G., Ogungbe I.V., Setzer W.N. (2017). Natural products as new treatment options for trichomoniasis: A molecular docking investigation. Sci. Pharm..

[130] Pan Y., Huang N., Cho S., MacKerell A.D. (2003). Consideration of molecular weight during compound selection in virtual target-based database screening. J. Chem. Inf. Comput. Sci..

[131] Huang N., Nagarsekar A., Xia G., Hayashi J., MacKerell A.D. (2004). Identification of non-phosphate-containing small molecular weight inhibitors of the tyrosine kinase p56 Lck SH2 domain via in silico screening against the pY + 3 binding site. J. Med. Chem..

[132] Carta G., Knox A.J.S., Lloyd D.G. (2007). Unbiasing scoring functions: A new normalization and rescoring strategy. J. Chem. Inf. Model..

